# rTMS ameliorates depressive‐like behaviors and regulates the gut microbiome and medium‐ and long‐chain fatty acids in mice exposed to chronic unpredictable mild stress

**DOI:** 10.1111/cns.14287

**Published:** 2023-06-02

**Authors:** Cui‐Hong Zhou, Yi‐Huan Chen, Shan‐Shan Xue, Qing‐Qing Shi, Lin Guo, Huan Yu, Fen Xue, Min Cai, Hua‐Ning Wang, Zheng‐Wu Peng

**Affiliations:** ^1^ Department of Psychiatry, Xijing Hospital Air Force Medical University Xi'an China; ^2^ Department of Psychiatry Chang'an Hospital Xi'an China

**Keywords:** CUMS, gut microbiota, medium‐ and long‐chain fatty acids, rTMS

## Abstract

**Introduction:**

Repetitive transcranial magnetic stimulation (rTMS) is a clinically useful therapy for depression. However, the effects of rTMS on the metabolism of fatty acids (FAs) and the composition of gut microbiota in depression are not well established.

**Methods:**

Mice received rTMS (15 Hz, 1.26 T) for seven consecutive days after exposure to chronic unpredictable mild stress (CUMS). The subsequent depressive‐like behaviors, the composition of gut microbiota of stool samples, as well as medium‐ and long‐chain fatty acids (MLCFAs) in the plasma, prefrontal cortex (PFC), and hippocampus (HPC) were evaluated.

**Results:**

CUMS induced remarkable changes in gut microbiotas and fatty acids, specifically in community diversity of gut microbiotas and PUFAs in the brain. 15 Hz rTMS treatment alleviates depressive‐like behaviors and partially normalized CUMS induced alterations of microbiotas and MLCFAs, especially the abundance of *Cyanobacteria*, *Actinobacteriota*, and levels of polyunsaturated fatty acids (PUFAs) in the hippocampus and PFC.

**Conclusion:**

These findings revealed that the modulation of gut microbiotas and PUFAs metabolism might partly contribute to the antidepressant effect of rTMS.

## Graphical abstract

rTMS (15 Hz, 1.26 T) alleviates depressive‐like behavior of CUMS‐treated mice and regulates the flora represented by *Cyanobacterium* and *Actinobaciota*. Meanwhile, it improves polyunsaturated fatty acids (PUFAs) in hippocampus and PFC, and also upregulates C20:4N6, C20:5N3 and C20:3N3 PUFAs in the plasma.

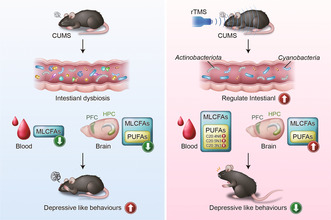

## 1 INTRODUCTION

Major depressive disorder (MDD) is a chronic progressive illness affecting millions globally with long‐term economic and social costs. Significantly, the emergence of the COVID‐19 pandemic in 2020 has created additional cases of MDD estimated at 50 million.^1^ The underlying pathophysiology of MDD was still explained mainly focusing on the dysfunction of monoamine neurotransmitters currently.^2^ However, antidepressants developed based on these therapies only alleviate symptoms in about 40–50% of patients with MDD,^3^ which even induces regrettable side effects, drug withdrawal problems, and a high recurrence rate.^4^ Therefore, it still needs to further explore the potential pathophysiology of MDD from a new perspective.

The microbiota–gut–brain axis is the bidirectional message transformation pathway, which plays an important role in the pathogenesis of MDD.^5^, ^6^, ^7^ There are multiple compositional differences in gut microbiota between patients with MDD and healthy controls,^8^, ^9^ and an altered gut metabolome contributes to depressive‐like behavior in rats.^10^ Moreover, prebiotics and probiotics have antidepressive effects partly through regulation of gut microecology^11^, ^12^ and fecal transplantation from patients with MDD, or mouse models of depression replicate depressive‐like behaviors in recipient germ‐free mice.^13^, ^14^ Besides, the vagus nerve system has been repeatedly identified as the most direct link between the brain and the gut microbiota.^15^, ^16^ For one thing, the vagus nerve is responsible for regulating metabolic homeostasis and feeding behavior, such as gastrointestinal motility and secretion functions. For another, much of the intestine‐ and microbiota‐related processes may influence the activity of the brain through the vagus nerve.^17^ Corresponding, a previous study found that subdiaphragmatic vagotomy significantly blocked the development of depressive‐like behaviors in mice induced by fecal microbiota transplantation (FMT) received from mice subjected to chronic social defeat stress,^18^ and vagus nerve stimulation (VNS) is a noninvasive alternative treatment for MDD, which was approved by Food and Drug Administration.^19^, ^20^ Nevertheless, the metabolites of gut microbiota also play causal roles in the development of MDD via the microbiota–gut–brain axis.^21^, ^22^ For instance, fatty acids (FAs) are key building blocks of lipids and essential components of the central nervous system (CNS).^23^ Beyond energy metabolism, FAs and their metabolites play essential roles such as neuroprotective molecules and anti‐inflammatory in MDD^24^ and the link between MDD and FAs metabolism in the plasma/serum has been also recognized.^25^, ^26^ Importantly, FAs have shown benefits to the brain as part of the direct or indirect link between “gut‐health” and “brain‐health” because they are naturally fermented or regulated by the gut microbiota.^24^ Previous studies found that gut microbiota can influence fatty acid production and metabolism,^27^, ^28^ and altered gut microbiota in depression were associated with disturbed peripheral and central lipids metabolism.^29^, ^30^ Accordingly, fecal microbial transplants from depression rat model could dysregulate fatty acids metabolism in recipient rats.^31^ Taken together, the regulation of gut microbiota and fatty acids metabolism might have translational applications in the treatment of depression.^32^, ^33^

Transcranial magnetic stimulation (TMS) is noninvasive neuromodulation technique. It has been successfully used as an important alternative therapy for depression.^34^ TMS could improve depressive symptoms and prevent the relapse of depressive episodes effectively in depression patients who have not responded to a full course of antidepressants.^35^, ^36^ In addition to the regulation of neuronal activity and functional connectivity in the brain, the influence of TMS on brain–gut communication has also attracted attention.^37^, ^38^, ^39^ A recent clinical study found that deep TMS treatment was revealed to be effective in modulating gut microbiota composition and food cravings in subjects with obesity.^40^ Another preclinical study also found that 10 Hz low‐intensity (13 mT) repetitive TMS (rTMS) treatment showed anti‐inflammatory and protective effects on the gut microbiome in chronic restraint stress‐treated rats.^41^ Although our recent study also found that rTMS with higher intensity (1.26 T) regulates brain lipid metabolism in both chronic unpredictable stress (CUS)‐treated rats and cuprizone‐treated mice,^42^, ^43^ little is known about the effect of rTMS on the metabolism of FAs as well as the influence of high‐intensity rTMS on the composition of gut microbiota in depression.

Considering the above, the present study used chronic unpredictable mild stress (CUMS)‐exposed mice, a well‐established mice model of depression^44^, ^45^ to determine the influence of rTMS with high‐frequency and high‐intensity (15 Hz, 1.26 T) on the composition of medium‐ and long‐chain fatty acids (MLCFAs) in the plasma, prefrontal cortex (PFC), and hippocampus (HPC) as well as the composition of gut microbiota. Furthermore, we also explored the relationship between changed gut microbiota, fatty acids, and depressive‐like behaviors.

## 2 MATERIALS AND METHODS

### 2.1 Animals

Male C57BL/6 mice aged at 8 weeks (18–22 g) used in this study were purchased from the Fourth Military Medical University Animal Center (Xi'an, China) and maintained at 20–25°C in a 12 h alternating light and dark cycle (lights on from 8:00 a.m. to 8:00 p.m.) with food and water available ad libitum. All experiments were approved by the Animal Use and Protection Committee of the Fourth Military Medical University and conducted in accordance with the National Institutes of Health Guide for the Care and Use of Laboratory Animals.

### 2.2 Experimental design

**FIGURE 1:**
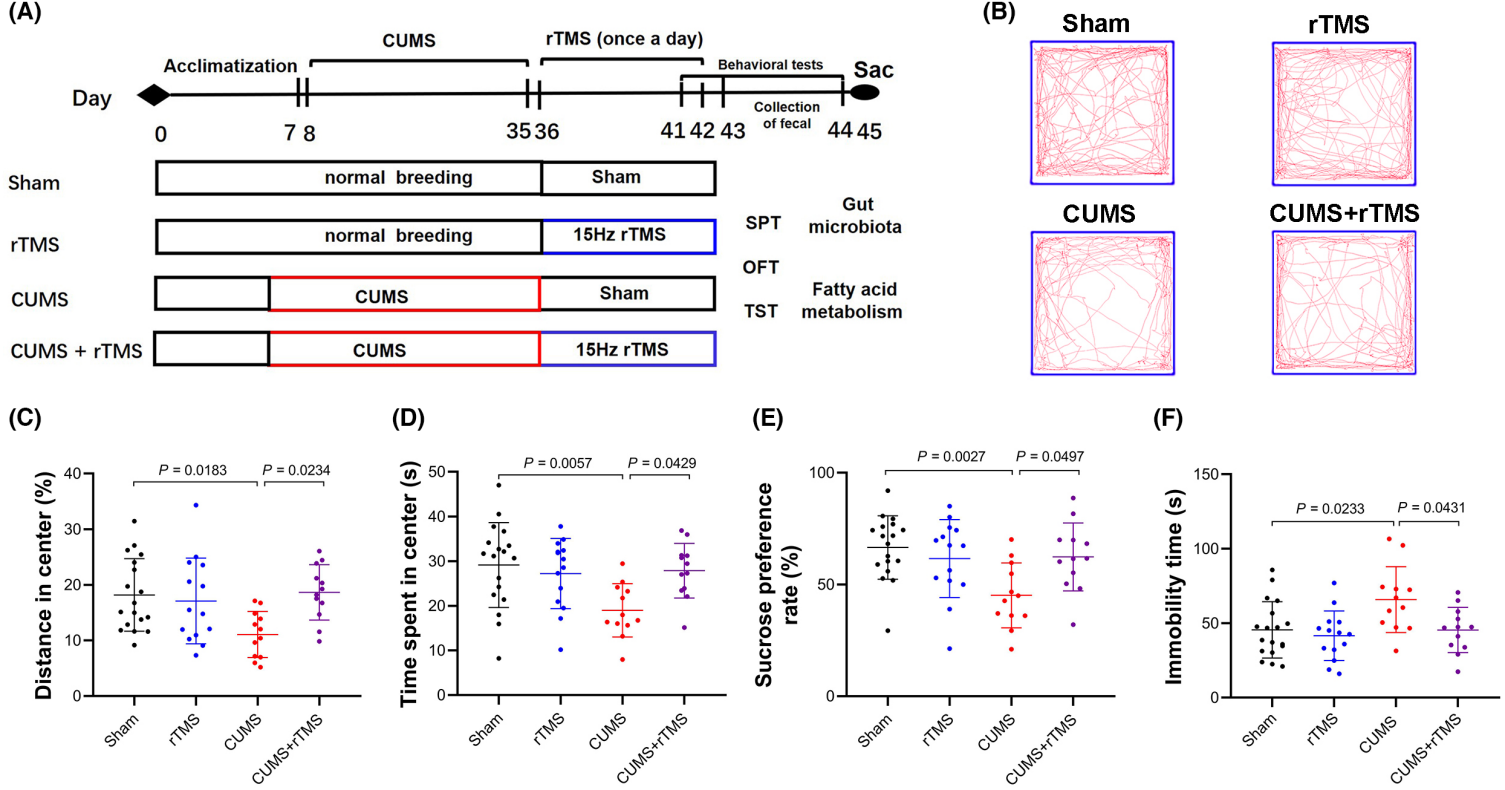
rTMS ameliorates depressive‐like behaviors in CUMS treated mice. (A) The experimental design. After 1 week of acclimatization, mice were subjected to CUMS or maintained in their home cages for 4 weeks, then rTMS or Sham stimulation was administered for 7 days. Behavioral testing and fecal collection were performed after the last rTMS intervention (the training phase for sucrose preference test was initiated on day 41), then the peripheral blood and brain tissues were collected for medium‐ and long‐chain fatty acids measurements. (B) Representative real‐time movement traces in the OFT for each group. (C) Percentage of distance traveled in central zone in the OFT. (D) Quantification of the time spent in center of the OFT. (E) The percentage of sucrose consumption in the SPT. (F) Immobility time measured in the TST. The dot represents one value from individual mice (sham group: *n* = 18; rTMS group: *n* = 14; CUMS group: *n* = 12; CUMS + rTMS group: *n* = 12); rTMS, repetitive transcranial magnetic stimulation; CUMS, chronic unpredicted mild stress; SPT, sucrose preference test; OFT, the open field test; TST, tail suspension test. Data was analyzed with two‐way analysis of variance (ANOVA) followed by a Bonferroni post‐hoc test for pairwise comparisons and detailed statistical information is provided in Table S1.

As shown in Figure 1A, mice were randomly distributed to the following four groups after 7 days of acclimatization: Sham (*n* = 18), rTMS (*n* = 14), CUMS (*n* = 12), and CUMS + rTMS (*n* = 12). Mice in the Sham and rTMS group were maintained in home cages for 4 weeks before subjected to sham rTMS or real rTMS (15 Hz, 1.26 Tesla) treatment for 7 days. Mice in the CUMS and CUMS + rTMS groups were subjected to CUMS for 28 days (day 8 to day35) and then received sham rTMS or real rTMS for 7 days (day 36 to day 42). The behavioral tests were conducted 24 h after the final intervention (The training phase for sucrose preference test was initiated after the 6th rTMS intervention (day 41), testing was performed on day 43). Feces were collected and stored in liquid nitrogen prior to behavioral testing. The plasma and brain tissue were collected and frozen in liquid nitrogen 24 h after the last behavioral test.

### 2.3 CUMS administration

The CUMS was performed as previously described.^46^ Briefly, mice were singly housed and subjected to various and repeated unpredictable stressors for 28 days as follows: (a) physical restraint (2 h); (b) continuous lighting (12 h); (c) food or water deprivation (24 h); (d) paired housing (24 h); (e) foot shocks (0.75 mA intensity, 2 s duration with 1 s intervals); (f) forced swim (8°C, 5 min); (g) wet bedding (24 h). Types of stimulation were randomly selected and applied daily, and each of the stressors was equally administered 3 or 4 times during the molding process.

### 2.4 rTMS treatment

rTMS was delivered at 15 Hz with a 3 s inter‐train interval and intensity of 1.26 Tesla using a circular coil (diameter, 23 mm; custom‐made YIRD, China) similar to our previous work.^47^ The daily stimulation consisted of 100 trains of 10 pulses and the trains were administered daily for 7 days at 1000 pulses per day. In the real stimulation groups, the coil was placed over the skull's vertex with the handle paralleling to the line of the animal's vertebral column. In the sham stimulation groups, the coil was held 10 cm above the head to ensure that the animal felt the vibrations produced by coil without any brain stimulation. Mouse was held to restrict movement by hand during the stimulation. Therefore, to exclude putative effects of nonspecific stress, all animals were habituated to the rTMS artifact noise sham stimulation procedure for 4 min every day for 7 days. There were no notable seizures or any behavioral changes throughout the treatment period in either real or sham stimulation.

### 2.5 Behavioral test

#### 2.5.1 Sucrose preference test

The sucrose preference test consisted of two sessions.^48^ During the training phase (Started on day 41), mice were allowed to consume water and 1% sucrose placed simultaneously freely for 24 h and then were deprived of water and food for 24 h. In the testing phase, mice were allowed to consume water and 1% sucrose, which were placed in pre‐weighed bottles freely for 2 h. The sucrose preference rate (%) = sucrose consumption/(sucrose consumption + water consumption).

#### 2.5.2 Open‐field test (OFT)

The open‐field test was performed according to a previous study.^49^ Briefly, animals were placed in the center of an open‐field box (40 cm × 40 cm × 40 cm), and activity was recorded for a period of 5 min, using the open‐field activity system (Open‐field Ji Liang Co. Ltd., Shanghai, China) and activity software (Top Scan, Clever Sys Inc., USA). The floor of the open field box was divided equally into 36 squares, including 16 central and 20 peripheral squares. The time spent and distance traveled in the central zone were recorded and measured.

#### 2.5.3 Tail suspension test (TST)

Tail suspension test was performed following a previous study.^50^ Mice were hung from a horizontal bar 60 cm above the floor by its tail for 6 minutes. Total immobility time was recorded and calculated without the first 2 minutes of the test session via automated tracking software (Freeze Scan, Clever Sys, Inc.). Criteria Immobility was set as being lack of skeletal movement for at least 1 s and mouse that crawled back up its tail was removed from the analysis.

### 2.6 Fecal sample collection and 16S rRNA Microbiome sequencing

Fecal samples were collected in a sterile cryotube between 7:00 to 11: 00 a.m. after the last intervention and quick‐frozen in liquid nitrogen immediately prior to analyses. Genomic DNA was extracted from fecal sample using E.Z.N.A. Stool DNA Kit (Omega Bio‐Tek, USA).^51^ The V3‐V4 hypervariable regions of bacterial 16S rRNA gene were amplified by polymerase chain reaction using primers 338F (5′‐ACTCCTACGGGAGGCAGCAG‐3′) and 806R (5′‐GGACTACHVGGGTWTCTAAT‐3′). After being extracted and quantified, amplicons were pooled in equimolar and paired‐end sequenced (2 × 250 bp) on an Illumina MiSeq PE300 platform according to the standard protocols in Sinotech Genome Technology Co. (Shanghai, China). Raw FASTQ files analysis was performed using USEARCH 8.0. Sequences were assigned to operational taxonomic units (OTUs) with UPARSE (version 7.1 http://drive5.com/uparse/) using 97% pairwise identity and the taxonomy were analyzed using the ribosomal database project classifier algorithm (http://rdp.cme.msu.edu/). And subsequent, Alpha Diversity Analysis, Principal coordinates analysis (PCoA), Linear discriminant analysis (LDA), PICRUSt2 function prediction analysis and correlation analysis were conducted at Majorbio Bio‐Pharm Technology Co., Ltd. (Shanghai, China) using QIIME software (version 1.9.1), R project Vegan package (version 2.5.3), R project ggplot2 package (version 2.2.1), KEGG database, and R project pheatmap package (version 3.3.1).

### 2.7 Blood and tissue collection

Mice were anesthetized with 2,2, 2‐tribromoethanol 24 h after the behavioral testing, and their blood and brain tissues were collected. Blood was collected from the mice eye pits into a 1.5 mL centrifuge tube pretreated with 20 μL of 100 U heparin sodium (Changzhou Qianhong Biopharma Co., Ltd. China), and then centrifuged at 1500 *g* for 15 min at 4°C. Then the plasma (supernatant) was collected and stored in liquid nitrogen prior to use. Subsequently, mice brains were removed and rinsed in ice‐cold phosphate buffered saline. The mPFC was isolated in a brain tank (68,713; RWD, Shenzhen, China), and the hippocampus was isolated under an anatomical microscope. All tissues were weighed and cut into pieces on ice and then were frozen and stored in liquid nitrogen. Finally, 8 samples of each group were used for fatty acid detection by Gas Chromatography–Mass Spectrometry (GC–MS).

### 2.8 Total fatty acids extraction

For brain tissue, samples were mixed with 2 mL of acidified methanol and incubated at 80°C for 30 min to achieve fatty‐acid methyl esterification. For plasm, 50 μL of each sample was mixed with 1 mL chloroform methanol (2:1 v/v) in a 2 mL glass centrifuge tube and ultrasonicated for 30 min. To achieve fatty‐acid esterification, 2 mL of 1% sulfuric acid in methanol was added to the supernatant, and then the mixture was incubated in water bath at 80°C for 30 min. Subsequently, both brain and plasm samples were extracted with 1 mL of hexane and washed with 5 mL of ddH_2_O. Next, 25 μL of internal standard (NU‐CHEK‐PREP methyl esterified fatty acids mixture added with methyl Salicylate) was mixed with 500 μL supernatant of the extract before GS‐MS.^52^

### 2.9 Medium‐ and long‐chain fatty acid measurements

Each 1 μL of extracted samples from brain tissue and plasm was analyzed by GC–MS in a single ion monitoring mode. Briefly, samples were separated and detected by Agilent 7890A/5975C system with Agilent DB‐WAX column (Agilent, 0.25 μm, 0.25 mm × 30 m). The initial column temperature remained at 50°C for 3 min, and then increased to 220°C at 10°C/min, and remained at 220°C for 20 min. Helium was used as the carrier gas, and mass spectrometry assay was performed using the Electron impact ion source (EI). The temperatures of the injection port, transmission line, and ion source were 280, 250, and 230°C, respectively. The stability and repeatability of the system were tested and evaluated through QC (quality‐control) sample in SIM (Selected ion Monitor) scanning mode throughout the experiment. Finally, MSD ChemStation was applied to analyze the mass data to determine the concentration of each compound.^52^

### 2.10 Statistical analysis

Behavioral and fatty acids data are presented as mean ± SD. Statistical analyses were conducted using GraphPad v.8.0 (GraphPad software) and R software package (http://www.R‐project.org/). The normal distribution of continuous data was detected by Shapiro–Wilk test. Data that did not satisfy normal distribution or homogeneity of variance were analyzed by nonparametric test (Kruskal–Wallis) and others were subjected to two‐way analysis of variance (ANOVA) followed by a Bonferroni post‐hoc test for pairwise comparisons. Pearson correlation was applied to analysis of the associations between fatty acids levels and behaviors, and the Spearman's rank correlation coefficient was applied to analysis of the correlations between behaviors and the top abundance 50 species at the genus level. All tests for significance were two‐tailed, and *p* < 0.05 was considered significant.

## 3 RESULTS

### 3.1 rTMS treatment ameliorates depressive‐like behaviors in CUMS mice

As shown in Figure 1 and Table S1, no identified differences were observed in the total distance (*F* = 0.189, *p* = 0.666; and *F* = 0.010, *p* = 0.921; respectively; data is not shown in the figure) or center distance (*F* = 2.788, *p* = 0.101; and *F* = 3.797, *p* = 0.057; respectively; Figure 1C) traveled in OFT either for the CUMS or for the rTMS treatment factors. Significant differences were observed in time spent in center (*F* = 5.083, *p =* 0.028; Figure 1D) of OFT and the sucrose preferences rate in the SPT (*F* = 6.189, *p =* 0.016; Figure 1E) for the CUMS factor, as well as immobility times displayed in the TST (Figure 1F) for both the CUMS factor (*F* = 5.879, *p =* 0.019) and rTMS factor (*F* = 5.982, *p =* 0.018). We also observed significant differences for the interaction factor in the percentage of center distance (*F* = 6.782, *p =* 0.012), time spent in center (*F* = 6.595, *p =* 0.013) as well as sucrose preference rate (*F* = 7.105, *p =* 0.010). Post hoc comparisons further showed that CUMS reduced the distance traveled in the central area and time spent in center of OFT and sucrose preferences rate in the SPT, but increased the immobility times in the TST significantly (CUMS vs. Sham, *p* < 0.05). rTMS treatment ameliorated the depressive‐like behavior of CUMS mice effectively, as evidenced by increasing central distance in the OFT, sucrose preferences rate in the SPT, and decreasing immobility time in the TST observed in rTMS + CUMS group (CUMS vs. CUMS + rTMS, *p* < 0.05), which are consistent with our previous study.^47^

### 3.2 rTMS changes the composition of gut microbiota in mice

**FIGURE 2:**
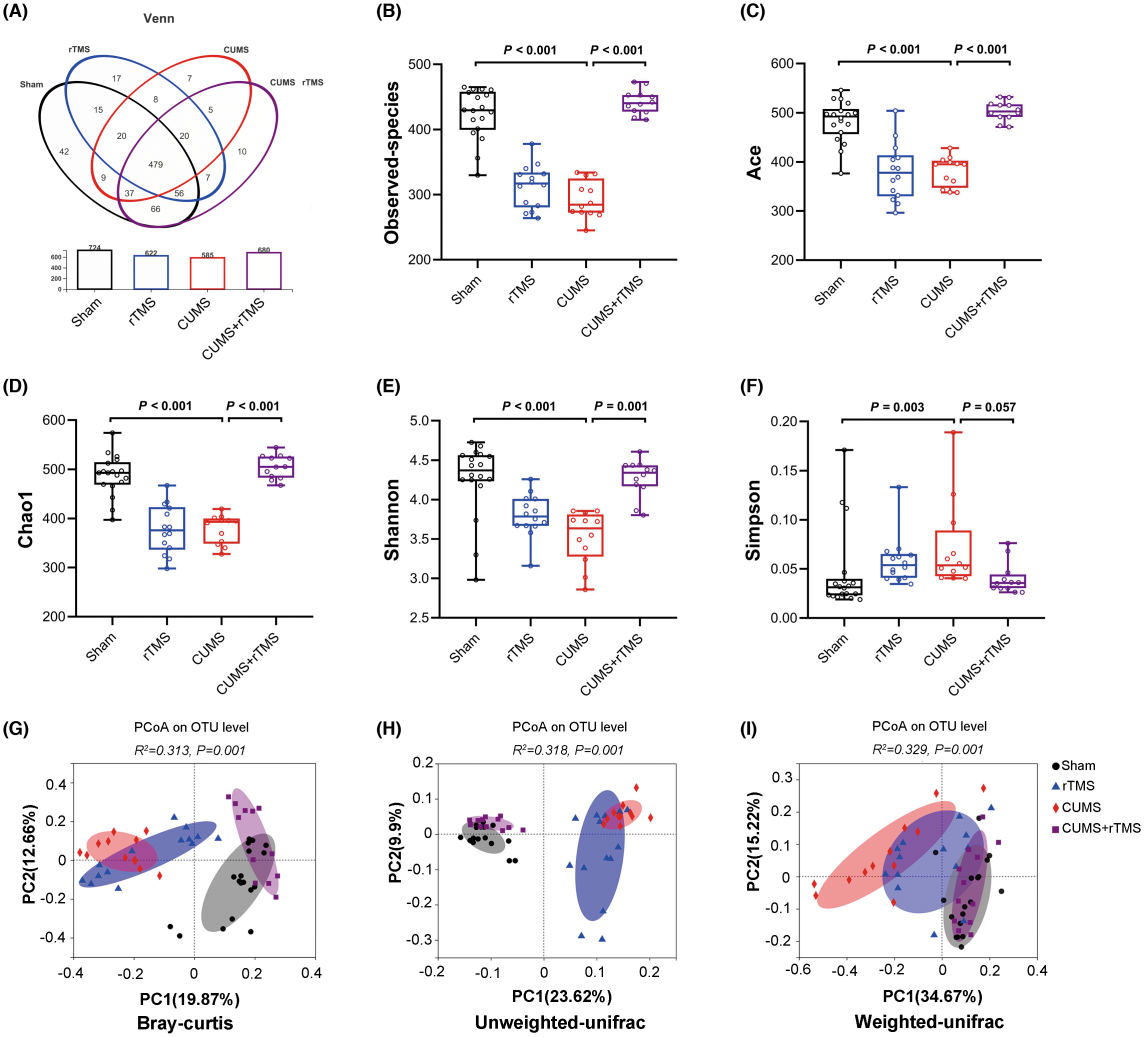
Differential gut microbial characteristics in mice of each group (sham group: *n* = 18; rTMS group: *n* = 14; CUMS group: *n* = 12; CUMS + rTMS group: *n* = 12). (A) The number of common and unique OUTs among the four groups is displayed by the Venn diagram and histogram. (B–F) Alpha diversity analysis index, including the (B) observed species (*H* = 40.268, *p* < 0.001), (C) Ace index (*H* = 34.421, *p* < 0.001), (D) chao1 index (*H* = 38.750, *p* < 0.001), (E) Shannon index (*H* = 27.362, *p* < 0.001), and (F) Simpson index (*H* = 18.144, *p* < 0.001). (G–I) PCoA plots of bacterial beta‐diversity on the basis of (G) Bray curtis, (H) Unweighted UniFrac distance and (I) Weighted UniFrac distance. The circle represents one value from individual mice (B–F). rTMS, repetitive transcranial magnetic stimulation; CUMS, chronic unpredicted mild stress; OUT, Operational taxonomic unit; PCoA, Principal coordinates analysis. Nonparametric test (Kruskal‐Wallis) was used in B–F.

We obtained a total of 1,117,341,014 bases and 2,582,388 high‐quality 16S rRNA gene sequences from 56 fecal samples. After downstream analysis, 924,726 sequences and 724 species‐level OTUs were obtained from the Sham group, 597,869 sequences and 622 OTUs from the rTMS group, 522,695 sequences and 585 species‐level OTUs from the CUMS group, and 537,098 sequences and 680 OTUs from the CUMS + rTMS group (Figure 2A). There was a significant difference in OTUs numbers between each group (*H* = 40.268, *p* < 0.001, Figure 2B). The α‐diversity values were compared, and significant differences were observed in the indices of Ace (*H* = 34.421, *p* < 0.001, Figure 2C), Chao1 (*H* = 38.750, *p* < 0.001, Figure 2D), Shannon (*H* = 27.362, *p* < 0.001, Figure 2E) and Simpson (*H* = 18.144, *p* < 0.001, Figure 2F) among the four groups. Post hoc comparisons revealed that the indices of Ace, Chao1, and Shannon were decreased in the CUMS group relative to the Sham group (CUMS vs. Sham, *p* = 0.001, *p* < 0.001 and *p* < 0.001, respectively), and rTMS treatment significantly increased the levels of Ace, Chao1 and Shannon in CUMS‐treated mice (CUMS + rTMS vs. CUMS, *p* < 0.001, *p* < 0.001 and *p* = 0.001, respectively). Additionally, the indices of Simpson were higher in the CUMS group when compared to the Sham group (*p* = 0.003), but rTMS treatment did not reverse this change induced by CUMS (CUMS + rTMS vs. CUMS, *p* = 0.057). Moreover, β‐diversity analysis was performed and fecal microbiomes among each group were divided into clusters according to microbial communities' composition by Bray‐Curtis (*r* ^2^ = 0.313, *p* = 0.001), unweighted UniFrac (*r* ^2^ = 0.318, *p* = 0.001), and weighted UniFrac (*r* ^2^ = 0.329, *p* = 0.001) analyses.

**FIGURE 3:**
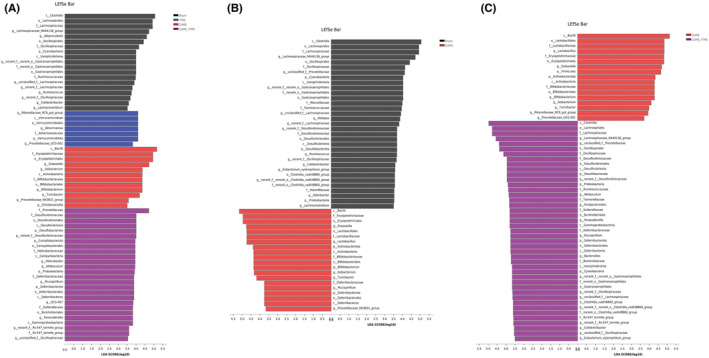
Differential taxonomic composition of gut microbiota in mice of each group (sham group: *n* = 18; rTMS group: *n* = 14; CUMS group: *n* = 12; CUMS + rTMS group: *n* = 12). (A) LDA score showed significant bacterial differences between these four groups based on the LEfSe and LDA analyses. (B) LDA score showed significant bacterial differences between the Sham and CUMS group. (C) LDA score showed significant bacterial differences between the CUMS and CUMS + rTMS groups. Only taxa with an LDA significance threshold >3.5 was presented. rTMS, repetitive transcranial magnetic stimulation; CUMS, chronic unpredicted mild stress; LDA, Linear discriminant analysis; LEfSe, Linear discriminant analysis effect size.

We validated differences in taxonomic composition between the four groups through linear discriminant analysis (LDA) and effect size (LEfSe) analysis (Figure 3A). We found that the relative abundance of phylum *Cyanobacteria*; class *Clostridia* and *Vampirivibrionia*; order *Lachnospirales*, *Oscillospiraceae*, *Gastranaerophilales*; family *Lachnospiraceae*, *Oscillospiraceae*, *norank_o_Gastranaerophilales*, *Ruminococcaceae*, and genus *Lachnospiraceae_NK3A136_group*, *Alloprevotella*, *norank_f_norank_o_Gastranaerophilales*, *norank_f_Lachnospiraceae*, *Ruminococcus*, *norank_f_Oscillospiraceae*, *Colidextribacter*, and *Lachnoclostridium* were enriched in the Sham group. The abundance of phylum *Verrucomicrobiota*, class *Verrucomicrobiae*, order *Verrucomicrobiales*, family *Akkermansiaceae*, and genus *Rikenellaceae_RC9_gut_group*, *Akkermansia* and *Prevotellaceae_UCG‐001* were enriched in the rTMS group. The abundance of class *Bacilli*, *Actinobacteria*; order *Bifidobacteriales*, family *Erysipelotrichaceae*, *Bifidobacteriaceae*, and genus *Dubosiella*, *Ileibacterium*, *Bifidobacterium*, *Turicibacter*, *Prevotellaceae_NK3B31_group*, and *Christensenella* were enriched in the CUMS group. The abundance of phylum *Desulfobacterota*, *Campilobacterota*, *Proteobacteria*, *Deferribacterota*; class *Desulfovibrionia*, *Campylobacteria*, *Deferribacteres*, *Gammaproteobacteria*; order *Desulfovibrionales*, *Campylobacterales*, *Deferribacterales*, *Burkholderiales*; family *Prevotellaceae*, *Desulfovibrionaceae*, *Helicobacteraceae*, *Deferribacteraceae*, *Sutterellaceae*, *Rs‐E47_termite_group*; genus *norank_f_Desulfovibrionaceae*, *Helicobacter*, *Allobaculum*, *Mucispirillum*, *UCG‐007*, *Parasutterella*, *norank_f_Rs‐E47_termite_group*, and *unclassified_f_Oscillospiraceae* were enriched in the CUMS + rTMS group.

### 3.3 Microbiota signatures specific for CUMS and rTMS treatment

In order to observe the effect of CUMS on the composition of fecal microbiota in mice, we compared the difference in gut microbiota between the Sham and the CUMS group. As shown in Figure 3B, the abundance of *Cyanobacteria*, *Desulfobacterota*, and *Proteobacteria* was enriched in the Sham group, while *Actinobacteriota* and *Deferribacterota* were enriched in the CUMS group at the phylum level. Meanwhile, the abundance of *Lachnospiraceae*, *Oscillospiraceae*, *norank_o_Gastranaerophilales*, *Rikenellaceae*, *Ruminococcaceae*, *Desulfovibrionaceae*, *norank_o_Clostridia_vadinBB60_group*, and *Marinifilaceae* was enriched in Sham group, whereas *Erysipelotrichaceae*, *Lactobacillaceae*, *Bifidobacteriaceae*, and *Deferribacteraceae* were enriched in the CUMS group at the family level. Moreover, we identified 14 genera that were abundant in the Sham group, whereas 7 genera were abundant in the CUMS group.

Furthermore, the differential microbial compositions between the CUMS and CUMS + rTMS were also analyzed to investigate microbial signatures discriminating rTMS treatment. We found that, at the phylum level, *Firmicutes* and *Actinobacteriota* were enriched in the CUMS group, whereas *Desulfobacterota*, *Proteobacteria*, *Deferribacterota*, and *Cyanobacteria* were enriched in the CUMS + rTMS group. At the family level, *Lactobacillaceae*, *Erysipelotrichaceae*, and *Bifidobacteriaceae* were enriched in the CUMS group, while *Lachnospiraceae*, *Oscillospiraceae*, *Desulfovibrionaceae*, *Ruminococcaceae*, *Tannerellaceae*, *Sutterellaceae*, *Deferribacteraceae*, *Bacteroidaceae*, *norank_o_Gastranaerophilales*, *norank_o_Clostridia_vadinBB60_group*, and *Rs‐E47_termite_group* were consistently higher in the CUMS + rTMS group. Finally, we identified 7 genera that were abundant in the CUMS group and 16 genera were abundant in the CUMS + rTMS group (Figure 3C). Notably, 14 genera altered in CUMS‐treated mice (9 increased and 5 decreased) were restored after rTMS treatment.

**FIGURE 4:**
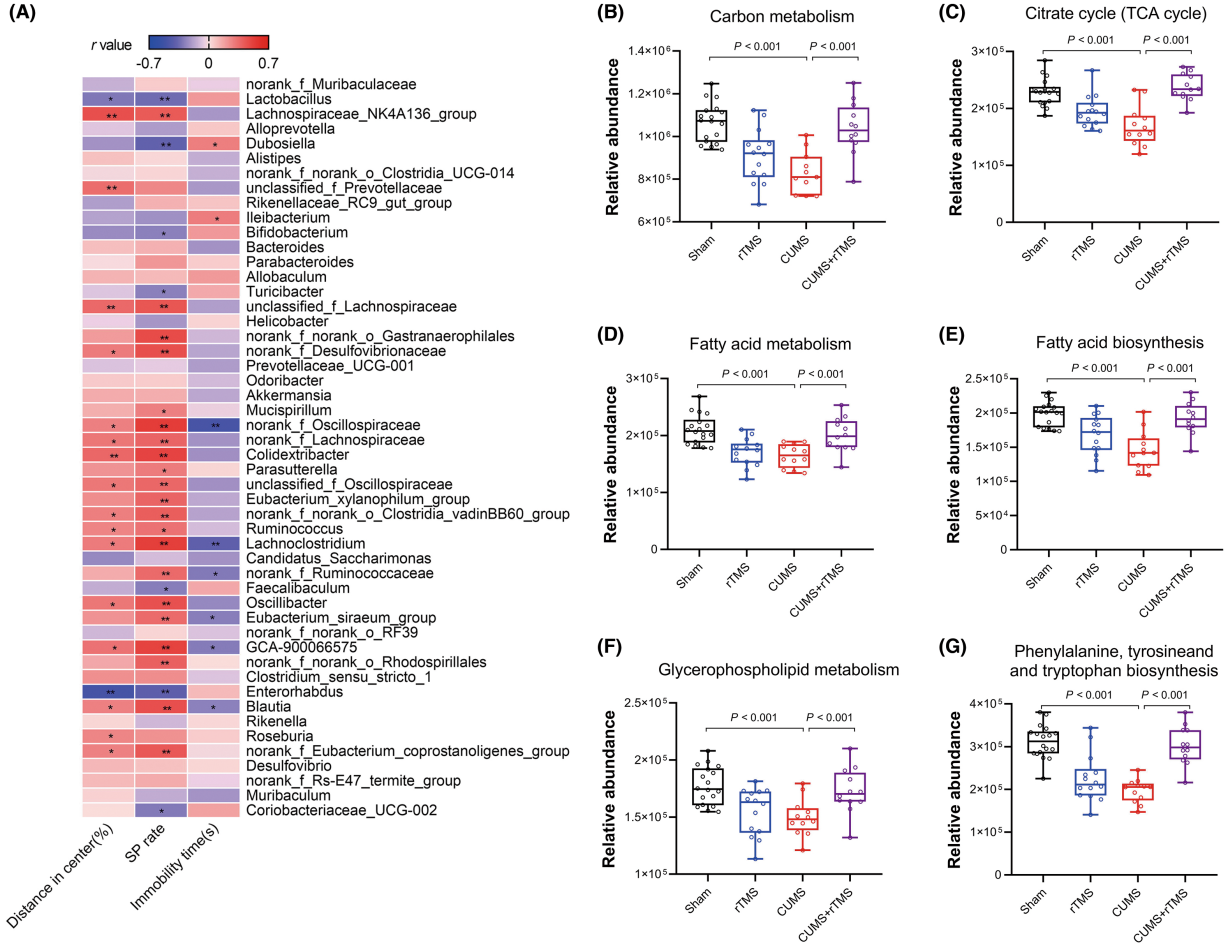
Correlation between depressive‐like behaviors and the differential gut microbiota and differences in gene function abundance between the four groups (sham group: *n* = 18; rTMS group: *n* = 14; CUMS group: *n* = 12; CUMS + rTMS group: *n* = 12). (A)The correlation between depressive‐like behaviors and the differential gut microbiota was analyzed at the genus level by Spearman's rank correlation coefficients, *p < 0.05; **p < 0.01, detailed statistical information is provided in Table S2. (B–G) the relative abundance of 6 KEGG pathways, (B) carbon metabolism, (C) citrate cycle (TCA cycle), (D) fatty acid metabolism, (E) fatty acid biosynthesis, (F) glycerophospholipid metabolism, and (G) phenylalanine, tyrosine, and tryptophan biosynthesis. The circle represents one value from individual mice. KEGG, Kyoto encyclopedia of genes and genomes; rTMS, repetitive transcranial magnetic stimulation; CUMS, chronic unpredicted mild stress; SP, sucrose preference. Two‐way analysis of variance (ANOVA) followed by a Bonferroni post‐hoc test for pairwise comparisons was used in date from B–G, detailed statistical information is provided in Table S3.

The correlation between depressive‐like behaviors and the differential gut microbiota at the genus level showed that the percentage of distance traveled in the center area was negatively correlated with the abundance of *Enterorhabdus* and *Lactobacillus*, but positively correlated with the abundance of 16 bacteria such as *unclassified_f_prevotellaceae*, *Colidextribacter* and *Lachnospiraceae_NK4A136_group* (*p* < 0.05). The percentage of sucrose preference rate was negatively correlated with the abundance of 7 bacteria such as *Lactobacillus*, *Enterorhabdus*, and *Dubosiella*, but positively correlated with the abundance of 21 bacteria such as *Lachnospiraceae_NK4136_group*, *Eubacterium_xylanophilum_group*, and *Lachnoclostridium* (*p* < 0.05). Moreover, the immobility time in TST was negatively correlated with the abundance of 6 bacteria, but positively correlated with the abundance of *Dubosiella* and *Ileibacterium*. (*p* < 0.05) (Figure 4A and Table S2).

### 3.4 Predicting the gene function of gut microbiota using KEGG

To further understand the functional information related to changes in the gut microbiota, functional prediction of important bacterial taxa among the four groups was achieved using PICRUSt2. A total of 245 pathways of level 3 were found to differ in functional abundance among the four groups through the Kyoto encyclopedia of genes and genomes (KEGG) database (*p* < 0.05), including most lipid metabolism, amino acid metabolism and Carbohydrate metabolism. Interestingly, significant differences were observed in the functional abundance levels of fatty acid metabolism (*F* = 5.217, *p* = 0.026) and fatty acid biosynthesis (*F* = 5.922, *p* = 0.018) for the CUMS factor (Table S3). We also observed significant differences for the interaction factor in the functional abundance levels of carbon metabolism, citrate cycle (TCA cycle), fatty acid metabolism, fatty acid biosynthesis, glycerophospholipid metabolism and phenylalanine, tyrosine and tryptophan biosynthesis. Post hoc comparisons further revealed that functional abundance levels of carbon metabolism, citrate cycle (TCA cycle), fatty acid metabolism, fatty acid biosynthesis, glycerophospholipid metabolism and phenylalanine, tyrosine and tryptophan biosynthesis were significantly reduced in CUMS‐treated mice (CUMS vs. Sham, *p* < 0.01), which were effectively normalized after rTMS treatment (CUMS + rTMS vs. CUMS, *p* < 0.01) (Figure 4B–G and Table S3).

### 3.5 Effect of rTMS on MLCFAs in the plasma

**FIGURE 5:**
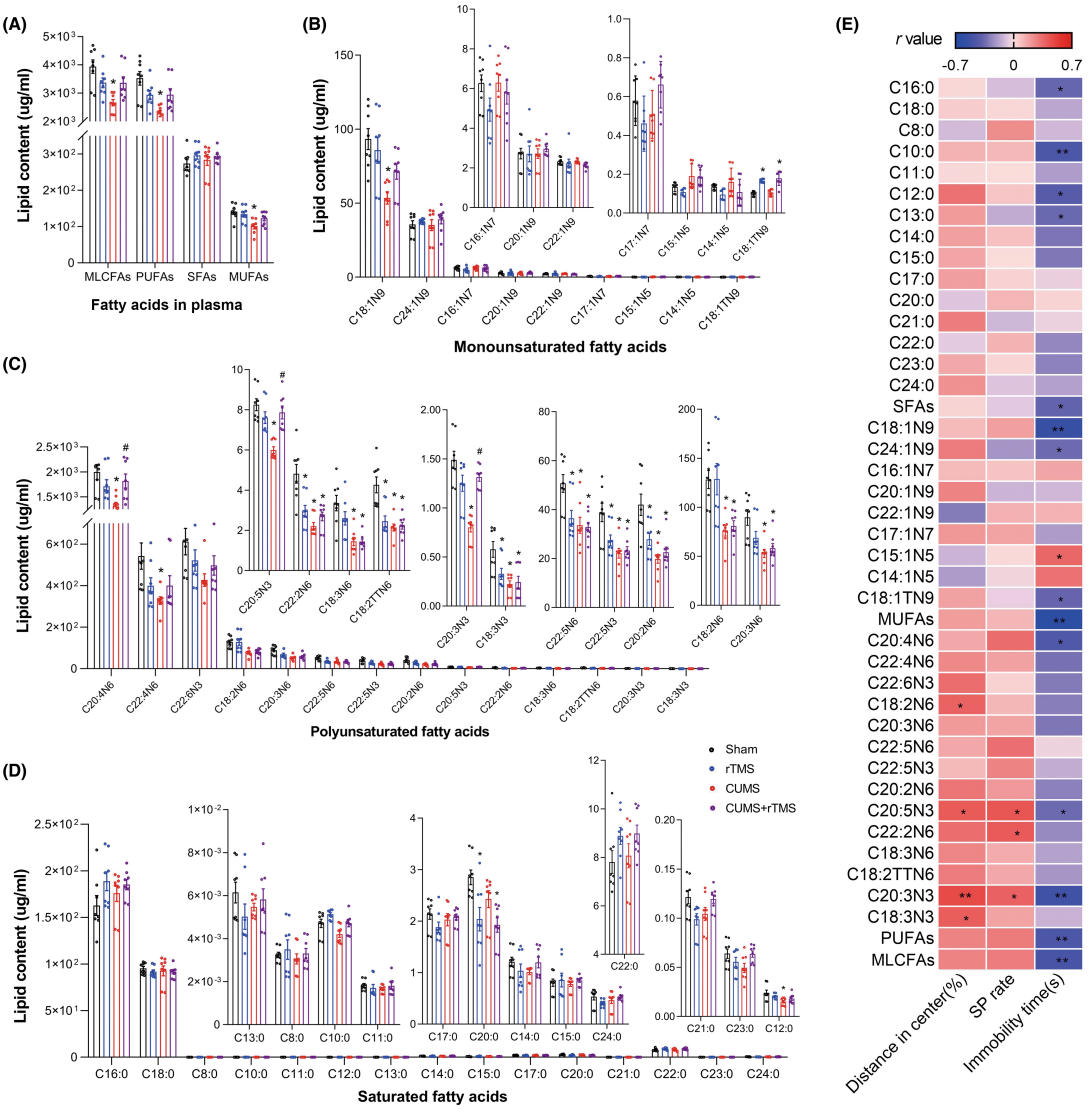
Groupwise alterations in the medium‐ and long‐chain fatty acid profiles in plasma. (A) Total MLCFAs, SFAs, MUFAs, and PUFAs concentration, (B) MUFA species, (C) PUFA species, (D) SFA species. (E) Analysis of correlations between depressive‐like behaviors and MLCFAs in plasma. The circle represents one value from individual mice (*n* = 8 in each group). MLCFAs, Medium‐ and long‐chain fatty acids; SFAs, Saturated fatty acids; MUFAs, Monounsaturated fatty acids; PUFAs, Polyunsaturated fatty acids; C8:0, Caprylic acid; C10:0, Capric acid; C11:0, Undecanoic acid; C12:0, Lauric acid; C13:0, Tritridecanoin; C14:0, Myristic acid; C15:0, Pentadecanoic acid; C16:0, Palmitic acid; C17:0, Heptadecanoic acid; C18:0, Stearic acid; C20:0, Arachidic acid; C21:0, Heneicosanoic acid; C22:0, Behenic acid; C23:0, Tricosanoic acid; C24:0, Lignoceric acid; C14:1N5, Myristoleic acid; C15:1N5, Pentadecenic acid; C16:1N7, Palmitoleic acid; C17:1N7, Margaric acid; 18:1N9, Oleic acid; C18:1TN9, Elaidic acid; C20:1N9, Eicosaenoic acid; C22:1N9, Erucic acid; C24:1N9, Nervonic acid; C18:2N6, Linoleic acid; C18:2TTN6, Linolelaidic acid; C20:2N6, Eicosatrienoic acid; C22:2N6, Docosadienoic acid; C18:3N6, Gamma‐linolenic acid; C18:3N3, α‐linolenic acid; C20:3N6, Gamma dihomo linoleic acid; C20:3N3, Eicosanotrienoic acid; C20:4N6, Arachidonic acid; C22:4N6, Docosatetraenoic acid; C22:5N6, Docosapentaenoic acid; C20:5N3, Eicosapentaenoic acid (EPA); C22:5N3, Docosa‐pentaenoic acid (DPA); C22:6N3, Docosahexaenoic acid (DHA); rTMS, repetitive transcranial magnetic stimulation; CUMS, chronic unpredicted mild stress; SP, sucrose preference. Two‐way analysis of variance (ANOVA) followed by a Bonferroni post‐hoc test for pairwise comparisons was used in (A‐D). *p < 0.05 vs. Sham; ^*#*^ *p* < *0.05* vs. CUMS; detailed statistical information is provided in Table S4; Pearson correlation was used in (E), *p < 0.05; **p < 0.01, detailed statistical information is provided in Table S5.

To identify the effects of CUMS and rTMS on fatty acid metabolism in peripheral blood, targeted metabolomic analysis was employed to evaluate the medium‐ and long‐chain fatty acids (MLCFAs) changes in plasma samples between groups (*n* = 8). We identified 38 medium‐ and long‐chain fatty acids in the plasma of each group, including 15 saturated fatty acids (SFAs), 9 monounsaturated fatty acids (MUFAs) and 14 polyunsaturated fatty acids (PUFAs). As shown in Figure 5 and Table S4, we observed significant differences for the CUMS factor in the concentrations of the following: total MLCFAs levels (*F* = 11.181, *p =* 0.002), total PUFAs (*F* = 10.964, *p =* 0.003) and 14 PUFAs such as C20:4N6, C18:3N3 and C18:3N6, total MUFAs and 2 MUFAs (C18:1N9, C15:1N5), and 2 SFAs (C10:0 and C12:0). In addition, we observed significant differences for the rTMS treatment factor in the concentrations of the following: 4 PUFAs such as C22:5N6, C20:5N3 and C22:2N6, and 2 MUFAs (C14:1N5 and C18:1TN9), and 3 SFAs (C10:0, C20:0 and C22:0). Consistently, significant differences were also observed for the interact factor in the concentrations of the following: total MLCFAs, total PUFAs and 11 PUFAs such as C20:4N6, C22:4N6 and C22:5N6, C17:1N7, and 2 SFAs (C21:0 and C23:0). Post hoc comparisons revealed that mice in the CUMS group exhibited significantly decreased levels of PUFAs, MUFAs, total MLCFAs C18:1N9, 13 PUFAs such as C20:4N6, C22:4N6, and C20:5N3 as well as C12:0 (CUMS vs. Sham, *p* < 0.05). rTMS treatment effectively increased the levels of C20:4N6, C20:5N3, and C20:3N3 (CUMS + rTMS vs. CUMS, *p* < 0.05) in CUMS‐treated mice. However, the changes in MUFAs, SFAs and other 10 PUFAs such as C22:4N6, C22:2N6, and C18:3N3 induced by CUMS in the plasma were not normalized after rTMS treatment (Figure 5A–D). Correlation analysis (Figure 5E and Table S5) showed that the percentage of distance traveled in the center squares of OFT was positively correlated with levels of C18:2N6, C20:5N3, C20:3N3 and C18:3N3 in the plasma. The sucrose preferences rate was also positively correlated with levels of C20:5N3, C22:2N6, and C20:3N3. The immobility time in TST was negatively correlated with levels of C16:0, C10:0, C12:0, C13:0 and total SFAs, C18:1N9, C24:1N9, C18:1TN9 and total MUFAs, C20:4N6, C20:5N3, C20:3N3 and total PUFAs as well as total lipids, but positively correlated with the level of C15:1N5 in the plasma. These results suggested that CUMS inhibits the level of MLCFAs, mainly MUFAs and PUFAs in the plasma, whereas rTMS can only restore several PUFAs.

### 3.6 Effect of rTMS on MLCFAs in the hippocampus and prefrontal cortex

**FIGURE 6:**
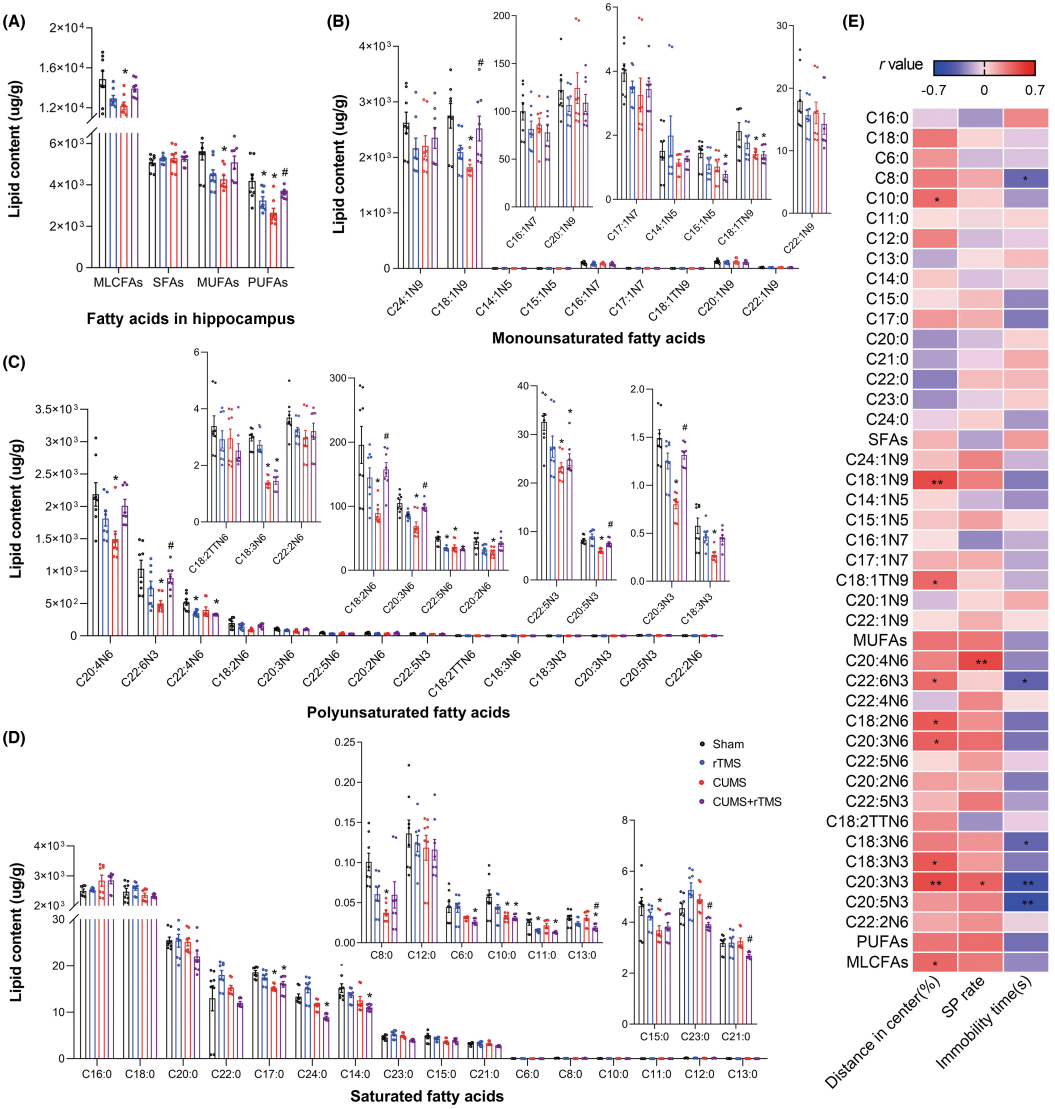
Groupwise alterations in the medium‐ and long‐chain fatty acid profiles in the hippocampus. (A) Total MLCFAs, SFAs, MUFAs, and PUFAs concentration, (B) MUFA species, (C) PUFA species, (D) SFA species. (E) Analysis of correlations between depressive‐like behaviors and MLCFAs in the hippocampus. The circle represents one value from individual mice (*n* = 8 in each group). MLCFAs, medium‐ and long‐chain fatty acids; SFAs, saturated fatty acids; MUFAs, monounsaturated fatty acids; PUFAs, polyunsaturated fatty acids; C6:0, Caproic acid; C8:0, Caprylic acid; C10:0, Capric acid; C11:0, Undecanoic acid; C12:0, Lauric acid; C13:0, Tritridecanoin; C14:0, Myristic acid; C15:0, Pentadecanoic acid; C16:0, Palmitic acid; C17:0, Heptadecanoic acid; C18:0, Stearic acid; C20:0, Arachidic acid; C21:0, Heneicosanoic acid; C22:0, Behenic acid; C23:0, Tricosanoic acid; C24:0, Lignoceric acid; C14:1N5, Myristoleic acid; C15:1N5, Pentadecenic acid; C16:1N7, Palmitoleic acid; C17:1N7, Margaric acid; 18:1N9, Oleic acid; C18:1TN9, Elaidic acid; C20:1N9, Eicosaenoic acid; C22:1N9, Erucic acid; C24:1N9, Nervonic acid; C18:2N6, Linoleic acid; C18:2TTN6, Linolelaidic acid; C20:2N6, Eicosatrienoic acid; C22:2N6, Docosadienoic acid; C18:3N6, Gamma‐linolenic acid; C18:3N3, α‐linolenic acid; C20:3N6, Gamma dihomo linoleic acid; C20:3N3, Eicosanotrienoic acid; C20:4N6, Arachidonic acid; C22:4N6, Docosatetraenoic acid; C22:5N6, Docosapentaenoic acid; C20:5N3, Eicosapentaenoic acid (EPA); C22:5N3, Docosa‐pentaenoic acid (DPA); C22:6N3, docosahexaenoic acid (DHA); rTMS, repetitive transcranial magnetic stimulation; CUMS, chronic unpredicted mild stress; SP, sucrose preference. Two‐way analysis of variance (ANOVA) followed by a Bonferroni post‐hoc test for pairwise comparisons was used in (A–D), detailed statistical information is provided in Table S6. Pearson correlation was used in (E), *p < 0.05; **p < 0.01, detailed statistical information is provided in Table S7.

**FIGURE 7:**
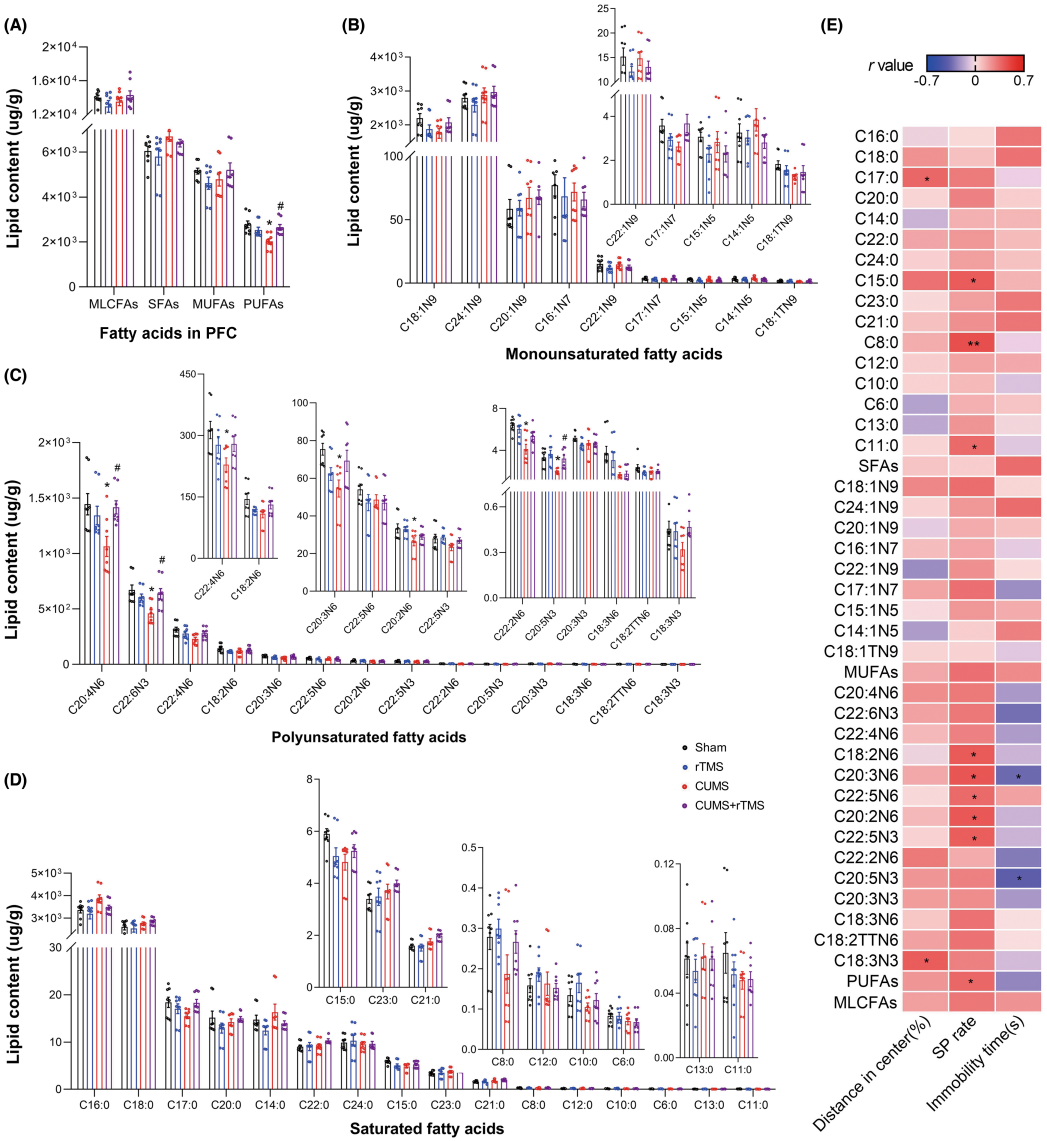
Groupwise alterations in the medium‐ and long‐chain fatty acid profiles in prefrontal cortex. (A) Total MLCFAs, SFAs, MUFAs, and PUFAs concentration, (B) MUFA species, (C) PUFA species, (D) SFA species. (E) Analysis of correlations between depressive‐like behaviors and MLCFAs in the prefrontal cortex. The circle represents one value from individual mice (*n* = 8 in each group). MLCFAs, medium‐ and long‐chain fatty acids; SFA, saturated fatty acid; MUFA, monounsaturated fatty acids; PUFA, polyunsaturated fatty acids; C6:0, Caproic acid; C8:0, Caprylic acid; C10:0, Capric acid; C11:0, Undecanoic acid; C12:0, Lauric acid; C13:0, Tritridecanoin; C14:0, Myristic acid; C15:0, Pentadecanoic acid; C16:0, Palmitic acid; C17:0, Heptadecanoic acid; C18:0, Stearic acid; C20:0, Arachidic acid; C21:0, Heneicosanoic acid; C22:0, Behenic acid; C23:0, Tricosanoic acid; C24:0, Lignoceric acid; C14:1N5, Myristoleic acid; C15:1N5, Pentadecenic acid; C16:1N7, Palmitoleic acid; C17:1N7, Margaric acid; 18:1N9, Oleic acid; C18:1TN9, Elaidic acid; C20:1N9, Eicosaenoic acid; C22:1N9, Erucic acid; C24:1N9, Nervonic acid; C18:2N6, Linoleic acid; C18:2TTN6, linolelaidic acid; C20:2N6, Eicosatrienoic acid; C22:2N6, Docosadienoic acid; C18:3N6, Gamma‐linolenic acid; C18:3N3, α‐linolenic acid; C20:3N6, Gamma dihomo linoleic acid; C20:3N3, Eicosanotrienoic acid; C20:4N6, Arachidonic acid; C22:4N6, Docosatetraenoic acid; C22:5N6, Docosapentaenoic acid; C20:5N3, Eicosapentaenoic acid (EPA); C22:5N3, Docosa‐pentaenoic acid (DPA); C22:6N3, docosahexaenoic acid (DHA); rTMS, repetitive transcranial magnetic stimulation; CUMS, chronic unpredicted mild stress; SP, sucrose preference. Two‐way analysis of variance (ANOVA) followed by a Bonferroni post‐hoc test for pairwise comparisons was used in (A–D), detailed statistical information is provided in Table S8. Pearson correlation was used in (E), *p < 0.05; **p < 0.01, detailed statistical information is provided in Table S9.

To investigate the effect of rTMS on brain fatty acid metabolism, medium‐ and long‐chain fatty acid changes in the hippocampus (Figure 6) and prefrontal cortex (PFC) (Figure 7) were also detected. As shown in Figure 6A–D and Table S6, we observed significant differences for the CUMS factor in the concentrations of the following in hippocampus: total PUFAs and 9 PUFAs such as C22:4N6, C18:2N6 and C20:3N3, 2 MUFAs (C15:1N5 and C18:1TN9), 10 SFAs such as C17:0, C8:0, and C10:0. In addition, we observed significant differences for the rTMS treatment factor in the concentrations of the following: 3 PUFAs (C22:4N6, C22:5N6 and C20:5N3), C15:1N5, and 4 SFAs such as C11:0, C13:0, and C14:0. Consistently, significant differences were also observed for the interact factor in the concentrations of the following: total MLCFAs, total PUFAs, and 9 PUFAs such as C22:5N6, C22:5N3 and C18:2N6, total MUFAs and C18:1N9, and 5 SFAs such as C8:0, C21:0, and C24:0.

Post hoc comparisons revealed that concentrations of MLCFAs, MUFAs, and PUFAs in the hippocampus were decreased in CUMS‐treated mice (CUMS vs. Sham, *p* < 0.05). However, rTMS treatment only reversed changes in total PUFAs induced by CUMS (CUMS + rTMS vs. CUMS, *p* < 0.05). Moreover, CUMS treatment decreased levels of C18:1N9 and C18:1TN9, and 11 PUFAs such as C20:4N6, C18:3N6 and C20:3N3, and 3 SFAs (C8:0, C17:0 and C15:0) in the hippocampus (CUMS vs. Sham, *p* < 0.05). rTMS treatment increased levels of C18:1N9, 5 PUFAs including C22:6N3, C18:2N6, C20:3N6, C20:5N3 and C20:3N3 but decreased levels of 3 SFAs (C13:0, C23:0 and C21:0) in the hippocampus of CUMS‐treated mice (CUMS + rTMS vs. CUMS, *p* < 0.05). Correlation analysis (Figure 6E and Table S7) showed that the percentage of distance traveled in the center squares of OFT was positively correlated with levels of C10:0, C18:1N9, C18:1TN9, and 5 PUFAs (C22:6N3, C18:2N6, C20:3N6, C18:3N3, C20:3N3), and total fatty acids in the hippocampus. The sucrose preferences rate was also positively correlated with levels of C20:4N6 and C20:3N3. The immobility time in TST was negatively correlated with levels of C8:0, C22:6N3, C18:3N6, C20:3N3 and C20:5N3 in the hippocampus.

In terms of PFC (Figure 7A–D and Table S8), there are significant differences for the CUMS factor in concentrations of following: total PUFAs and 7 PUFAs such as C22:6N3, C22:4N6 and C20:2N6, total SFAs and 3 SFAs (C6:0, C17:0 and C21:0). We observed significant differences for the rTMS treatment factor in the concentrations of C20:5N3 and C12:0. Moreover, we also observed significant differences for interaction factor in the concentrations of following: total PUFAs and 6 PUFAs such as C22:6N3, C22:4N6 and C22:2N6, 2 MUFAs (C18:1N9 and C17:1N7), and 2 SFAs (C10:0 and C15:0). Post hoc comparison further showed that CUMS treatment decreased levels of total PUFAs, C20:4N6, C22:6N3, C22:4N6, C20:3N6, C20:2N6, C22:2N6 and C20:5N3 in the PFC (CUMS vs. Sham, *p* < 0.05). rTMS treatment reversed the changes of PUFAs, C20:4N6, C22:6N3 and C20:5N3 that induced by CUMS (CUMS + rTMS vs. CUMS, *p* < 0.05). Correlation analysis further showed that the percentage of distance traveled in the center squares of OFT was positively correlated with levels of C17:0 and C18:3N3. Meanwhile, the sucrose preferences rate was also positively correlated with levels of 3 SFAs (C15:0, C8:0 and C11:0), and 5 PUFAs (C18:2N6, C20:3N6, C22:5N6, C20:2N6 and C22:5N3) as well as total PUFAs in the PFC. The immobility time in TST was negatively correlated with levels of C20:3N6 and C20:5N3 in the PFC (Figure 7E and Table S9). These results suggested that CUMS remarkably reduces the concentration of MUFAs and PUFAs in the hippocampus and PUFAs in the PFC, which was also partially restored by rTMS treatment.

## 4 DISCUSSION

In the current study, we performed integrative analysis to assess the effect of high‐frequency rTMS treatment on the composition of gut microbiota and medium‐ and long‐chain fatty acids (MLCFAs) in a CUMS‐induced mice model of depression. CUMS induced remarkable changes in gut microbiotas and fatty acids, specifically in community diversity of gut microbiotas and PUFAs in the brain. 15 Hz rTMS treatment alleviates depressive‐like behaviors and partially normalized CUMS induced alterations of microbiotas and MLCFAs, especially the abundance of *Cyanobacteria*, *Actinobacteriota*, and levels of PUFAs in the hippocampus and PFC. These findings elucidated that dysfunction of gut microbiota and MLCFAs metabolism might be participate in the pathogenesis of MDD, and modulation of gut microbiotas and PUFAs metabolism in the hippocampus and PFC might partly contribute to the antidepressant effect of rTMS.

Bidirectional gut–brain axis communication has been widely accepted.^53^, ^54^ Brain can modulate the gastrointestinal tract and enteric nervous system via the parasympathetic and sympathetic branches of the autonomic nervous system and the HPA axis directly,^55^ and can influence the enteric microbiota indirectly by altering its microenvironment.^56^ As a result, neuromodulation technology could affect the composition and function of gut microbiota.^57^ Conversely, gut microbiota can regulate many aspects of host physiology thus influencing brain development and function.^58^ Although the involvement of gut microbiota in the pathogenesis of depression has been largely reported, including the relationship between the characteristic gut microbiota, diversity of gut microbiota and the severity of depressive symptoms in patients with depression as well as the effect of antidepressants on the composition of gut microbiota,^59^, ^60^ the results of characteristic microbiota and diversity identified in different studies are not completely consistent. For instance, there is no consensus regarding whether the gut microbiota richness and diversity changes in depression models. A recent work found that the Shannon and Simpson indices showed no significant difference whereas the Chao1 and ACE indices was decreased,^61^ while another work showed that the diversity of the fecal microbiome but not the richness was reduced in CUMS treated rats.^62^ Similarly, the community diversity and richness estimators were not changed in CUMS‐treated mice in a previous study,^63^ but these estimators were reduced in another study.^64^ Inconsistent with the previous study,^41^ the present study showed that the alpha diversity in the CUMS model was significantly reduced and rTMS treatment reversed these changes effectively (including Ace, Chao1, and Shannon). In addition, the beta diversity analysis showed a different community composition of the gut microbiome between the CUMS‐ and rTMS‐treated group, indicating the potential effect of rTMS on the diversity of intestinal flora.

Furthermore, our results also found that the microbial composition in CUMS‐treated mice was obviously different from Sham group. For instance, *Cyanobacteria* and *Proteobacteria* phylum were enriched in Sham group, whereas *Actinobacteriota* was enriched in CUMS mice (CUMS vs. Sham). *Cyanobacteria* are considered beneficial bacteria and are well known for the production of pharmacologically important metabolites that exhibit antidepressive properties, and researchers have found that antidepressant treatment promoted the abundance of *Cyanobacteria* phylum in a stress rat model.^65^, ^66^ Meanwhile, a high abundance of *Actinobacteriota*, which are extremely versatile producers of bioactive natural products including antibiotics immunosuppressants and antiviral compounds, has been observed in depression subjects.^67^ Although a high abundance of *Proteobacteria* is thought to be associated with inflammation in gut, enrichment of *Proteobacteria* in depression is still controversial. A previous study reported that *Proteobacteria* is enriched MDD,^68^ while other researchers found a lower abundance of this phylum in patients with depression.^9^ Importantly, rTMS treatment increased the relative abundance of *Cyanobacteria* and *Proteobacteria* phylum and decreased the level of *Actinobacteriota* significantly in CUMS‐treated mice (CUMS + rTMS vs. CUMS), suggesting that the regulation of phyla *Cyanobacteria*, *Proteobacteria*, and *Actinobacteriota* at phylum level might be involved in the antidepressive like effects. Of note, we also found that rTMS treatment significantly restored the relative abundances of 14 genera that altered in the CUMS‐induced mice. Among these genera, the abundance of 6 genera (*norank_f_norank_o_Clostridia_vadinBB60_group, norank_f_Desulfovibrionaceae, norank_f_Oscillospiraceae, Lachnospiraceae_NK4A136_group, unclassified_f_Lachnospiraceae, and Colidextribacter*) was positively correlated with both the percentage of distance traveled in the center area and sucrose preference rate. The abundance of *Lactobacillus*, *Dubosiella*, *Bifidobacterium*, and *Turicibacter* was negatively correlated with sucrose preference rate. Moreover, the abundance of *Ileibacterium* was positively whereas *norank_f_Oscillospiraceae* was negatively correlated with the immobility time in TST. Due to these, microbiotas also have been reported to be involved in neurotransmitters and intestinal inflammation,^69^ the regulation of these microbiotas might be involved in the antidepressive effect of rTMS. Besides, the comorbidity of anxiety and depression is very common in clinical practice. Indeed, CUMS can not only induce depressive‐like behavior but also anxiety‐like behavior,^70^ and fecal microbiota transplantation from CUMS‐treated mice donors also affects anxiety‐like and depression‐like behavior in recipient mice via the gut microbiota–inflammation–brain axis.^71^ Moreover, the anti‐anxiety effect of rTMS has been extensively reported,^72^ and the add‐on rTMS treatment can improve anxiety symptoms in patients with depression comorbid with anxiety.^73^ On the other hand, both patients with anxiety disorder and anxiety‐like animal models exhibit gut microbiota imbalance.^74^, ^75^ Together, intestinal dysbacteriosis might be a common pathogenesis both in anxiety and depression. However, the co‐pathogenic bacteria of anxiety and depression, as well as the regulatory effect of TMS on the microbiota of anxiety animal models, still need further exploration.

Accumulating evidence indicated that depression is linked to abnormal lipid metabolism, especially fatty acids and their metabolites, which are the key messengers in the bidirectional communication between the gut microbiota and the brain.^10^, ^76^ In consistent, the present study found that 245 Kyoto encyclopedia of genes and genomes (KEGG) pathways varied significantly in relative abundance among the groups and the relative abundance of energy metabolism (carbon metabolism and TCA cycle), phenylalanine, tyrosine and tryptophan biosynthesis, glycerophospholipid metabolism, and fatty acid metabolism and biosynthesis pathways were decreased in CUMS‐treated mice, all of which were normalized after rTMS treatment. It suggested that the regulation of metabolic function of bacteria may be related to the antidepressant effect of rTMS.

Fatty acids (FAs) play an important role in regulating energy homeostasis, neurotransmission and signaling pathways, and ultimately affects emotional behavior.^24^, ^77^ FAs are classified as SFAs, MUFAs, and PUFAs. Previous studies found that intake of SFAs induces depressive‐like behavior in rodents,^78^, ^79^ whereas intake of MUFAs might be benefit to brain function, such as the facilitation of neurotransmitter signal transduction.^80^, ^81^ In the present study, there is no significant difference in the total SFAs in the plasma, hippocampus, and PFC between each group. Meanwhile, total MUFAs were decreased in plasma and hippocampus in CUMS group, which could not be normalized after rTMS treatment. It suggests that SFAs may not be closely related to CUMS‐induced depressive‐like behaviors. Importantly, levels of oleic acid (18:1N9) were decreased after CUMS both in the plasma and hippocampus, and rTMS treatment only restored it in the hippocampus. Due to oleic acid can increase neural stem cell mitotic activity and drive hippocampal neurogenesis in mice^82^ and prolonged intake of oleic acid enriched diet in human reduces the risk of depression,^83^, ^84^ the increased oleic acid in the hippocampus might be involved in the antidepressant effect of rTMS.

Nevertheless, more studies focus on the involvement of PUFAs in depression.^85^ According to the position of the first double bond, PUFAs are distinguished into N3 and N6 PUFAs. The two essential PUFAs linoleic acid (18:2N6, LA) and alpha‐linolenic acid (18:3N3, ALA), which are mainly provided by diet and act as precursors of arachidonic acid (20:4N6, AA), eicosapentaenoic acid (20:5N3, EPA), and docosahexaenoic acid (22:6N3, DHA), regulate both the structure and the function of cells in the brain.^86^ Previous studies reported that decreased PUFAs in patients with depression^87^ and lower N3 PUFAs levels in baseline showed reduced response to standard antidepressants.^88^ Meanwhile, nutritional intervention with EPA or DHA provides beneficial anti‐inflammatory and anti‐depressant effects both in clinical and basic research.^89^, ^90^ Here, we found that levels of ALA and LA were decreased in the plasma and hippocampus, and the levels of EPA and AA were decreased in the plasma, hippocampus, and PFC, whereas level of DHA was decreased only in hippocampus and PFC in CUMS‐induced mice. Importantly, rTMS treatment restored levels of total PUFAs, including EPA and DHA in the hippocampus and PFC. Meanwhile, rTMS treatment also increased EPA in the plasma of CUMS‐treated mice effectively, suggesting that these N3 PUFAs were involved in the protective effect of rTMS. Furthermore, AA, a precursor for two main endocannabinoids‐ anandamide (AEA) and 2‐arachidonoylglycerol (2‐AG), plays a key role in modulating synaptic plasticity and neurotransmitter release in the brain.^91^, ^92^ Previous work found that serum AA was decreased in childhood and adolescent patients with depression,^93^ and a dysfunctional endocannabinoid system (ECS) in the CNS has also been increasingly implicated in the pathophysiology of depression.^94^ The present study found that rTMS increased level of AA in PFC of CUMS‐induced mice, indicating that rTMS might play a neuroprotective and antidepressant role by regulating AA metabolism to enhance endocannabinoid signaling. Moreover, our results also found that rTMS treatment reversed the level of eicosatrienoic acid (20:3N3) in the plasma and hippocampus of CUMS‐induced mice, which was negatively associated with the severity of depression. As eicosatrienoic acid is produced during arachidonic acid metabolism and plays a role in neuroprotective effects on the CNS,^95^ this PUFA may also involve in the antidepressant effects of rTMS, and more details remain to be determined. It cannot be ignored that the soluble epoxide hydrolase (sEH) is a key enzyme in the metabolism of PUFA and plays a key role in the inflammation that is involved in depression.^96^, ^97^ Meanwhile, epoxy fatty acids such as epoxyeicosatrienoic acids (EETs) and epoxyeicosapentaenoic acids (EDPs) have been found to exert neuroprotective effects through potent anti‐inflammatory actions.^98^ Unfortunately, the present study did not observe the content of sEH and epoxy fatty acids, which may further explain the antidepressant effect of TMS.

On the contrary, recent studies also found N3 supplementation is not enough to make recommendations in preventing depression symptoms^99^ and even nonclinically beneficial effect of N3 on depressive symptomology when compared to placebo.^100^ On the other hand, AA is also a substrate for the synthesis of the inflammatory mediators, such as prostaglandins (PGs) and leukotrienes (LTs). PGE2 mediates depression‐like behavior induced by repeated social defeat stress and PGD2 has been implicated in depression‐like behaviors induced by chronic stress.^101^ Moreover, a previous clinical study also found depressive symptoms were negatively correlated with serum AA levels.^102^ Furthermore, multiple studies suggested the measurement of the N6/N3 ratio rather than the content of N6 or N3 might be a useful indication for depressive symptoms.^103^, ^104^ This disparity might be related to the high individual variability in FAs composition together with the presence of confounders. The ability to control for potential effects of environment, diet, and lifestyle on the FAs and gut microbiome might be an important advantage of performing animal studies. However, little is known about this controversial result in animal research, which needs to be verified in future studies.

Finally, there are some limitations that should be noted. First, the underlying mechanisms of rTMS alter intestinal flora and fatty acid metabolism remains unclear. We speculate that the regulation of the brain‐gut axis may be one of the underlying mechanisms. Due to the subdiaphragmatic vagus nerve plays a crucial role in the crosstalk between the brain and gut microbiota,^105^, ^106^ and subdiaphragmatic vagotomy (SDV) blocks depression‐related behaviors in the antibiotic cocktail‐treated mice after ingestion of specific microbe.^18^, ^107^ It is necessary to observe the influence of SDV on the antidepressant effects as well as the regulation of gut microbiome and fatty acid metabolism of rTMS. Meanwhile, the effects of different patterns of rTMS on gut microbiota and fatty acids metabolism still need to clarify. Although the current study has confirmed the correlation analysis between changes either in gut microbiota or in fatty acids content and behavioral outcomes, the correlation between the gut microflora and fatty acids content in brain still needs to be investigated. Nevertheless, we did not consider the role of gender differences in CUMS and the antidepressive‐like effect of rTMS, which should also be elucidated in the future.

## 5 CONCLUSIONS

rTMS alleviates depressive‐like behaviors and partially rescues the gut microbial dysbiosis and disturbance of fatty acid metabolism. rTMS regulates the abundances of *Cyanobacteria*, *Proteobacteria*, and *Actinobacteriota* phylum, which are related to neurotransmitters and intestinal inflammation. In addition, rTMS regulates the levels of fatty acids, mainly belonging to PUFAs in plasma and brain tissues, especially increasing the levels of DAH, EPA in the hippocampus and PFC. Therefore, our results shed a light on the relationship between physical stimulation and metabolism and might provide new ideas for further research on the antidepressant effect of rTMS.

## CONFLICT OF INTEREST STATEMENT

The authors have no conflicts of interest to declare.

## Supporting information


Table S1

Table S2

Table S3

Table S4

Table S5

Table S6

Table S7

Table S8

Table S9

## Data Availability

The data that support the findings of this study are available from the corresponding author upon reasonable request.
