# Supplementary material for: rTMS ameliorates depressive‐like behaviors and regulates the gut microbiome and medium‐ and long‐chain fatty acids in mice exposed to chronic unpredictable mild stress
Source: CNS Neurosci Ther. 2023 Jun 2;29(11):3549–66. doi: 10.1111/cns.14287 (PMC10580350; doi:10.1111/cns.14287)
Supplement: Supplementary file 1 — Table S1 [file CNS-29-3549-s003.docx]

**Supplemental Table 1. The effect of CUMS and rTMS on behavior in mice**

| **Behavioral index** | **rTMS factor** | | **CUMS factor** | | **rTMS*CUMS** | |
| --- | --- | --- | --- | --- | --- | --- |
|  | F | *P* | F | *P* | F | *P* |
| Total distance in OFT | 0.010 | 0.921 | 0.189 | 0.666 | 0.456 | 0.502 |
| Distance in center (%) | 3.797 | 0.057 | 2.788 | 0.101 | 6.782 | 0.012 |
| Time spent in center (s) | 2.728 | 0.1046 | 5.083 | 0.028 | 6.595 | 0.013 |
| Sucrose preference rate (%) | 2.162 | 0.147 | 6.189 | 0.016 | 7.105 | 0.010 |
| Immobility time (TST) | 5.982 | 0.018 | 5.879 | 0.019 | 2.709 | 0.106 |
