# Supplementary material for: rTMS ameliorates depressive‐like behaviors and regulates the gut microbiome and medium‐ and long‐chain fatty acids in mice exposed to chronic unpredictable mild stress
Source: CNS Neurosci Ther. 2023 Jun 2;29(11):3549–66. doi: 10.1111/cns.14287 (PMC10580350; doi:10.1111/cns.14287)
Supplement: Supplementary file 2 — Table S2 [file CNS-29-3549-s009.docx]

**Supplementary Table 2. Correlation between depressive-like behaviors and gut microbiotas**

| Lipids | Distance in center (%) | | Sucrose preference rate (%) | | Immobility time (s) | |
| --- | --- | --- | --- | --- | --- | --- |
|  | r value | *P* value | r value | *P* value | r value | *P* value |
| G_norank_f_Muribaculaceae | -0.139 | 0.306 | 0.053 | 0.699 | -0.052 | 0.701 |
| g_Lactobacillus | -0.311 | 0.020 | -0.346 | 0.009 | 0.206 | 0.128 |
| g_Lachnospiraceae_NK4A136_group | 0.450 | 0.001 | 0.410 | 0.002 | -0.215 | 0.111 |
| g_Alloprevotella | -0.079 | 0.561 | -0.201 | 0.138 | 0.067 | 0.623 |
| g_Dubosiella | -0.230 | 0.088 | -0.382 | 0.004 | 0.287 | 0.032 |
| g_Alistipes | 0.080 | 0.560 | 0.017 | 0.899 | -0.148 | 0.276 |
| g_norank_f_norank_o_Clostridia_UCG-014 | -0.023 | 0.867 | 0.020 | 0.886 | 0.144 | 0.291 |
| g_unclassified_f_Prevotellaceae | 0.343 | 0.010 | 0.257 | 0.055 | -0.210 | 0.120 |
| g_Rikenellaceae_RC9_gut_group | -0.196 | 0.148 | 0.130 | 0.341 | 0.073 | 0.595 |
| g_Ileibacterium | -0.184 | 0.176 | -0.236 | 0.080 | 0.289 | 0.031 |
| g_Bifidobacterium | -0.250 | 0.063 | -0.287 | 0.032 | 0.204 | 0.132 |
| g_Bacteroides | 0.088 | 0.517 | 0.129 | 0.345 | -0.230 | 0.088 |
| g_Parabacteroides | 0.013) | 0.925 | 0.210 | 0.121 | 0.052 | 0.705 |
| g_Allobaculum | 0.122 | 0.370 | 0.105 | 0.442 | 0.200 | 0.140 |
| g_Turicibacter | -0.081 | 0.554 | -0.286 | 0.032 | 0.151 | 0.267 |
| g_unclassified_f_Lachnospiraceae | 0.357 | 0.007 | 0.429 | 0.001 | -0.194 | 0.151 |
| g_Helicobacter | -0.047 | 0.729 | -0.219 | 0.105 | 0.029 | 0.832 |
| g_norank_f_norank_o_Gastranaerophilales | 0.194 | 0.151 | 0.468 | <0.001 | -0.121 | 0.375 |
| g_norank_f_Desulfovibrionaceae | 0.298 | 0.026 | 0.449 | 0.001 | -0.174 | 0.199 |
| g_Prevotellaceae_UCG-001 | -0.090 | 0.509 | -0.064 | 0.637 | -0.203 | 0.134 |
| g_Odoribacter | 0.058 | 0.671 | 0.051 | 0.708 | -0.112 | 0.413 |
| g_Akkermansia | 0.145 | 0.286 | 0.138 | 0.309 | -0.166 | 0.222 |
| g_Mucispirillum | 0.137 | 0.316 | 0.279 | 0.037 | -0.044 | 0.746 |
| g_norank_f_Oscillospiraceae | 0.280 | 0.037 | 0.530 | <0.001 | -0.492 | <0.001 |
| g_norank_f_Lachnospiraceae | 0.311 | 0.020 | 0.374 | 0.005 | -0.230 | 0.089 |
| g_Colidextribacter | 0.363 | 0.006 | 0.498 | <0.001 | -0.238 | 0.077 |
| g_Parasutterella | 0.224 | 0.096 | 0.294 | 0.028 | 0.024 | 0.862 |
| g_unclassified_f_Oscillospiraceae | 0.307 | 0.021 | 0.367 | 0.005 | -0.235 | 0.081 |
| g_Eubacterium_xylanophilum_group | 0.242 | 0.072 | 0.374 | 0.005 | -0.165 | 0.225 |
| g_norank_f_norank_o_Clostridia_vadinBB60_group | 0.286 | 0.033 | 0.398 | 0.002 | -0.178 | 0.188 |
| g_Ruminococcus | 0.267 | 0.046 | 0.323 | 0.015 | -0.104 | 0.447 |
| g_Lachnoclostridium | 0.299 | 0.025 | 0.522 | <0.001 | -0.393 | 0.003 |
| g_Candidatus_Saccharimonas | -0.258 | 0.055 | -0.105 | 0.440 | -0.228 | 0.091 |
| g_norank_f_Ruminococcaceae | 0.150 | 0.270 | 0.354 | 0.007 | -0.306 | 0.022 |
| g_Faecalibaculum | -0.147 | 0.281 | -0.294 | 0.028 | 0.155 | 0.254 |
| g_Oscillibacter | 0.311 | 0.020 | 0.445 | 0.001 | -0.259 | 0.053 |
| g_Eubacterium_siraeum_group | 0.221 | 0.102 | 0.350 | 0.008 | -0.295 | 0.027 |
| g_norank_f_norank_o_RF39 | -0.117 | 0.389 | 0.026 | 0.849 | -0.089 | 0.513 |
| g_GCA-900066575 | 0.327 | 0.014 | 0.504 | 0.000 | -0.298 | 0.026 |
| g_norank_f_norank_o_Rhodospirillales | 0.161 | 0.235 | 0.387 | 0.003 | 0.004 | 0.975 |
| g_Clostridium_sensu_stricto_1 | 0.232 | 0.086 | 0.239 | 0.077 | -0.084 | 0.539 |
| g_Enterorhabdus | -0.496 | <0.001 | -0.396 | 0.002 | 0.105 | 0.441 |
| g_Blautia | 0.278 | 0.038 | 0.451 | <0.001 | -0.283 | 0.035 |
| g_Rikenella | 0.024 | 0.859 | -0.116 | 0.393 | 0.017 | 0.904 |
| g_Roseburia | 0.264 | 0.049 | 0.228 | 0.091 | 0.056 | 0.684 |
| g_norank_f_Eubacterium_coprostanoligenes_group | 0.278 | 0.038 | 0.422 | 0.001 | -0.024 | 0.859 |
| g_Desulfovibrio | 0.112 | 0.410 | 0.080 | 0.559 | 0.023 | 0.868 |
| g_norank_f_Rs-E47_termite_group | 0.104 | 0.448 | 0.150 | 0.271 | -0.047 | 0.730 |
| g_Muribaculum | 0.037 | 0.788 | -0.152 | 0.262 | -0.226 | 0.094 |
| g_Coriobacteriaceae_UCG-002 | 0.005 | 0.972 | -0.303 | 0.023 | 0.175 | 0.196 |
