# Supplementary material for: rTMS ameliorates depressive‐like behaviors and regulates the gut microbiome and medium‐ and long‐chain fatty acids in mice exposed to chronic unpredictable mild stress
Source: CNS Neurosci Ther. 2023 Jun 2;29(11):3549–66. doi: 10.1111/cns.14287 (PMC10580350; doi:10.1111/cns.14287)
Supplement: Supplementary file 3 — Table S3 [file CNS-29-3549-s006.docx]

**Supplementary Table 3. Effect of CUMS and rTMS on KEGG pathway**

| **KEGG pathway** | **rTMS factor** | | **CUMS factor** | | **rTMS*CUMS** | |
| --- | --- | --- | --- | --- | --- | --- |
|  | F | *P* | F | *P* | F | *P* |
| Carbon metabolism | 0.436 | 0.512 | 3.667 | 0.061 | 38.566 | <0.001 |
| Citrate cycle (TCA cycle) | 3.469 | 0.068 | 2.505 | 0.120 | 45.559 | <0.001 |
| Fatty acid metabolism | 1.511 | 0.225 | 5.217 | 0.026 | 49.338 | <0.001 |
| Fatty acid biosynthesis | 1.123 | 0.294 | 5.922 | 0.018 | 36.535 | <0.001 |
| Glycerophospholipid metabolism | 0.002 | 0.969 | 1.214 | 0.276 | 21.262 | <0.001 |
| Phenylalanine, tyrosine and tryptophan biosynthesis | 0.591 | 0.445 | 2.908 | 0.094 | 68.778 | <0.001 |
