# Supplementary material for: rTMS ameliorates depressive‐like behaviors and regulates the gut microbiome and medium‐ and long‐chain fatty acids in mice exposed to chronic unpredictable mild stress
Source: CNS Neurosci Ther. 2023 Jun 2;29(11):3549–66. doi: 10.1111/cns.14287 (PMC10580350; doi:10.1111/cns.14287)
Supplement: Supplementary file 4 — Table S4 [file CNS-29-3549-s005.docx]

**Supplementary Table 4. Effect of CUMS and rTMS on the concentration of MLCFAs in the plasma**

| **Fatty acids** | **rTMS factor** | | **CUMS factor** | | **rTMS*CUMS** | |
| --- | --- | --- | --- | --- | --- | --- |
|  | F | *P* | F | *P* | F | *P* |
| C20:4N6 | 0.732 | 0.400 | 5.083 | 0.032 | 9.545 | 0.004 |
| C22:4N6 | 0.619 | 0.438 | 5.542 | 0.026 | 5.548 | 0.026 |
| C22:6N3 | 0.043 | 0.837 | 4.482 | 0.043 | 2.640 | 0.115 |
| C18:2N6 | 0.071 | 0.792 | 25.031 | 0.000 | 0.049 | 0.826 |
| C20:3N6 | 1.680 | 0.206 | 15.198 | 0.001 | 4.603 | 0.041 |
| C22:5N6 | 5.945 | 0.021 | 11.304 | 0.002 | 4.827 | 0.036 |
| C22:5N3 | 4.155 | 0.051 | 17.137 | <0.001 | 6.039 | 0.020 |
| C20:2N6 | 3.977 | 0.056 | 23.215 | <0.001 | 8.807 | 0.006 |
| C20:5N3 | 5.000 | 0.033 | 13.285 | 0.001 | 21.045 | <0.001 |
| C22:2N6 | 4.988 | 0.034 | 21.702 | <0.001 | 14.639 | 0.001 |
| C18:3N6 | 1.860 | 0.183 | 29.871 | <0.001 | 1.780 | 0.193 |
| C18:2TTN6 | 10.380 | 0.003 | 19.109 | <0.001 | 12.601 | 0.001 |
| C20:3N3 | 4.106 | 0.052 | 21.318 | <0.001 | 30.721 | <0.001 |
| C18:3N3 | 3.617 | 0.068 | 13.148 | 0.001 | 4.981 | 0.034 |
| PUFAs | 0.045 | 0.834 | 10.964 | 0.003 | 11.337 | 0.002 |
| C18:1N9 | 0.627 | 0.435 | 17.094 | <0.001 | 3.757 | 0.063 |
| C24:1N9 | 1.204 | 0.282 | 0.024 | 0.878 | 0.098 | 0.757 |
| C16:1N7 | 3.069 | 0.091 | 0.779 | 0.385 | 0.686 | 0.415 |
| C20:1N9 | 0.156 | 0.695 | 0.200 | 0.658 | 0.210 | 0.650 |
| C22:1N9 | 1.675 | 0.206 | 0.202 | 0.656 | 0.188 | 0.668 |
| C17:1N7 | 0.149 | 0.702 | 2.047 | 0.164 | 8.881 | 0.006 |
| C15:1N5 | 1.108 | 0.302 | 19.029 | <0.001 | 0.526 | 0.474 |
| C14:1N5 | 6.536 | 0.016 | 0.932 | 0.343 | 0.084 | 0.773 |
| C18:1TN9 | 85.892 | <0.001 | 1.559 | 0.222 | 0.210 | 0.650 |
| MUFAs | 1.009 | 0.324 | 13.087 | 0.001 | 3.899 | 0.058 |
| C16:0 | 3.911 | 0.058 | 0.284 | 0.598 | 0.861 | 0.361 |
| C18:0 | 0.479 | 0.494 | 0.163 | 0.689 | 0.150 | 0.701 |
| C8:0 | 0.728 | 0.401 | 0.418 | 0.523 | 0.000 | 1.000 |
| C10:0 | 9.470 | 0.005 | 9.519 | 0.005 | 0.092 | 0.764 |
| C11:0 | 0.005 | 0.944 | 0.069 | 0.794 | 1.131 | 0.297 |
| C12:0 | 0.026 | 0.872 | 9.378 | 0.005 | 3.225 | 0.083 |
| C13:0 | 0.703 | 0.409 | 0.017 | 0.898 | 2.470 | 0.127 |
| C14:0 | 0.006 | 0.939 | 0.040 | 0.843 | 3.461 | 0.073 |
| C15:0 | 0.571 | 0.456 | 0.057 | 0.813 | 0.011 | 0.917 |
| C17:0 | 1.111 | 0.301 | 0.198 | 0.660 | 2.486 | 0.126 |
| C20:0 | 14.594 | 0.001 | 2.433 | 0.130 | 0.815 | 0.374 |
| C21:0 | 0.407 | 0.529 | 0.093 | 0.763 | 10.094 | 0.004 |
| C22:0 | 5.618 | 0.025 | 0.176 | 0.678 | 0.038 | 0.846 |
| C23:0 | 0.425 | 0.520 | 0.561 | 0.460 | 6.654 | 0.015 |
| C24:0 | 0.332 | 0.569 | 0.387 | 0.539 | 4.029 | 0.054 |
| SFAs | 2.068 | 0.162 | 0.097 | 0.758 | 0.354 | 0.557 |
| MLCFAs | 0.107 | 0.746 | 11.181 | 0.002 | 11.024 | 0.003 |
