# Supplementary material for: rTMS ameliorates depressive‐like behaviors and regulates the gut microbiome and medium‐ and long‐chain fatty acids in mice exposed to chronic unpredictable mild stress
Source: CNS Neurosci Ther. 2023 Jun 2;29(11):3549–66. doi: 10.1111/cns.14287 (PMC10580350; doi:10.1111/cns.14287)
Supplement: Supplementary file 5 — Table S5 [file CNS-29-3549-s002.docx]

**Supplementary Table 5. Correlation between depressive-like behaviors and levels of MLCFAs in the plasma**

| Fatty acids | Distance in center (%) | | Sucrose preference rate (%) | | Immobility time (s) | |
| --- | --- | --- | --- | --- | --- | --- |
|  | r value | *P* value | r value | *P* value | r value | *P* value |
| C16:0 | 0.018 | 0.922 | -0.098 | 0.593 | -0.368 | 0.038 |
| C18:0 | 0.047 | 0.797 | 0.019 | 0.919 | -0.168 | 0.358 |
| C8:0 | -0.109 | 0.554 | 0.240 | 0.186 | -0.114 | 0.536 |
| C10:0 | 0.124 | 0.498 | 0.108 | 0.556 | -0.465 | 0.007 |
| C11:0 | 0.013 | 0.943 | 0.006 | 0.974 | -0.198 | 0.277 |
| C12:0 | 0.331 | 0.064 | 0.072 | 0.696 | -0.420 | 0.017 |
| C13:0 | 0.197 | 0.281 | -0.154 | 0.401 | -0.359 | 0.044 |
| C14:0 | 0.182 | 0.319 | 0.083 | 0.650 | -0.317 | 0.077 |
| C15:0 | 0.166 | 0.363 | 0.006 | 0.976 | -0.311 | 0.084 |
| C17:0 | 0.188 | 0.304 | 0.032 | 0.862 | -0.044 | 0.811 |
| C20:0 | -0.079 | 0.668 | 0.118 | 0.521 | 0.029 | 0.873 |
| C21:0 | 0.290 | 0.107 | -0.118 | 0.519 | -0.041 | 0.823 |
| C22:0 | -0.065 | 0.725 | 0.123 | 0.501 | -0.265 | 0.143 |
| C23:0 | 0.157 | 0.391 | 0.020 | 0.915 | -0.281 | 0.119 |
| C24:0 | 0.225 | 0.215 | -0.082 | 0.654 | -0.185 | 0.309 |
| SFAs | 0.030 | 0.869 | -0.070 | 0.702 | -0.378 | 0.033 |
| C18:1N9 | 0.100 | 0.586 | 0.187 | 0.305 | -0.562 | 0.001 |
| C24:1N9 | 0.292 | 0.104 | -0.221 | 0.224 | -0.351 | 0.049 |
| C16:1N7 | 0.101 | 0.581 | 0.076 | 0.680 | 0.163 | 0.372 |
| C20:1N9 | 0.273 | 0.131 | -0.128 | 0.486 | -0.117 | 0.523 |
| C22:1N9 | -0.297 | 0.098 | 0.131 | 0.476 | 0.126 | 0.492 |
| C17:1N7 | 0.208 | 0.253 | 0.173 | 0.345 | -0.202 | 0.267 |
| C15:1N5 | -0.124 | 0.499 | 0.011 | 0.952 | 0.365 | 0.040 |
| C14:1N5 | -0.202 | 0.266 | -0.031 | 0.868 | 0.323 | 0.071 |
| C18:1TN9 | 0.180 | 0.323 | -0.050 | 0.787 | -0.372 | 0.036 |
| MUFAs | 0.193 | 0.289 | 0.114 | 0.533 | -0.629 | <0.001 |
| C20:4N6 | 0.154 | 0.399 | 0.347 | 0.051 | -0.448 | 0.010 |
| C22:4N6 | 0.255 | 0.158 | 0.181 | 0.322 | -0.328 | 0.067 |
| C22:6N3 | 0.343 | 0.055 | 0.036 | 0.847 | -0.303 | 0.092 |
| C18:2N6 | 0.381 | 0.031 | 0.112 | 0.542 | -0.289 | 0.108 |
| C20:3N6 | 0.200 | 0.273 | 0.171 | 0.349 | -0.315 | 0.080 |
| C22:5N6 | 0.162 | 0.376 | 0.336 | 0.060 | -0.034 | 0.853 |
| C22:5N3 | 0.103 | 0.573 | 0.274 | 0.129 | -0.144 | 0.431 |
| C20:2N6 | 0.313 | 0.082 | 0.204 | 0.264 | -0.284 | 0.115 |
| C20:5N3 | 0.410 | 0.020 | 0.430 | 0.014 | -0.352 | 0.048 |
| C22:2N6 | 0.341 | 0.056 | 0.408 | 0.021 | -0.257 | 0.155 |
| C18:3N6 | 0.264 | 0.145 | 0.148 | 0.420 | -0.182 | 0.319 |
| C18:2TTN6 | 0.293 | 0.104 | 0.159 | 0.384 | -0.219 | 0.229 |
| C20:3N3 | 0.469 | 0.007 | 0.382 | 0.031 | -0.522 | 0.002 |
| C18:3N3 | 0.395 | 0.025 | 0.173 | 0.342 | -0.133 | 0.468 |
| PUFAs | 0.270 | 0.135 | 0.297 | 0.098 | -0.462 | 0.008 |
| Total MLCFAs | 0.272 | 0.132 | 0.291 | 0.107 | -0.490 | 0.004 |
