# Supplementary material for: rTMS ameliorates depressive‐like behaviors and regulates the gut microbiome and medium‐ and long‐chain fatty acids in mice exposed to chronic unpredictable mild stress
Source: CNS Neurosci Ther. 2023 Jun 2;29(11):3549–66. doi: 10.1111/cns.14287 (PMC10580350; doi:10.1111/cns.14287)
Supplement: Supplementary file 6 — Table S6 [file CNS-29-3549-s001.docx]

**Supplementary Table 6. Effect of CUMS and rTMS on the concentration of MLCFAs in the hippocampus**

| **Fatty acids** | **rTMS factor** | | **CUMS factor** | | **rTMS*CUMS** | |
| --- | --- | --- | --- | --- | --- | --- |
|  | F | *P* | F | *P* | F | *P* |
| C20:4N6 | 0.281 | 0.600 | 3.462 | 0.073 | 11.290 | 0.002 |
| C22:6N3 | 0.217 | 0.645 | 4.026 | 0.055 | 13.181 | 0.001 |
| C22:4N6 | 12.696 | 0.001 | 4.294 | 0.048 | 2.069 | 0.161 |
| C18:2N6 | 0.237 | 0.630 | 7.145 | 0.012 | 11.589 | 0.002 |
| C20:3N6 | 1.523 | 0.227 | 5.764 | 0.023 | 28.422 | <0.001 |
| C22:5N6 | 17.828 | <0.001 | 10.030 | 0.004 | 4.611 | 0.041 |
| C20:2N6 | 0.028 | 0.869 | 1.557 | 0.222 | 14.072 | 0.001 |
| C22:5N3 | 1.227 | 0.277 | 14.188 | 0.001 | 4.359 | 0.046 |
| C18:2TTN6 | 2.047 | 0.164 | 1.778 | 0.193 | 0.004 | 0.953 |
| C18:3N6 | 0.760 | 0.391 | 156.225 | <0.001 | 1.869 | 0.183 |
| C18:3N3 | 0.456 | 0.505 | 10.586 | 0.003 | 8.408 | 0.007 |
| C20:3N3 | 4.106 | 0.052 | 21.318 | <0.001 | 30.721 | <0.001 |
| C20:5N3 | 12.254 | 0.002 | 29.379 | <0.001 | 1.692 | 0.204 |
| C22:2N6 | 0.237 | 0.631 | 2.224 | 0.147 | 2.359 | 0.136 |
| PUFAs | 0.000 | 0.999 | 6.660 | 0.015 | 17.307 | <0.001 |
| C24:1N9 | 0.705 | 0.408 | 0.373 | 0.546 | 2.597 | 0.118 |
| C18:1N9 | 0.017 | 0.897 | 2.177 | 0.151 | 15.669 | <0.001 |
| C14:1N5 | 0.754 | 0.393 | 2.497 | 0.125 | 0.269 | 0.608 |
| C15:1N5 | 4.412 | 0.045 | 6.881 | 0.014 | 0.214 | 0.648 |
| C16:1N7 | 2.575 | 0.120 | 1.135 | 0.296 | 0.395 | 0.535 |
| C17:1N7 | 0.151 | 0.700 | 1.311 | 0.262 | 0.884 | 0.355 |
| C18:1TN9 | 1.210 | 0.281 | 10.062 | 0.004 | 1.034 | 0.318 |
| C20:1N9 | 2.102 | 0.158 | 0.044 | 0.836 | 0.000 | 0.987 |
| C22:1N9 | 1.748 | 0.197 | 1.111 | 0.301 | 0.028 | 0.869 |
| MUFAs | 0.292 | 0.593 | 1.501 | 0.231 | 10.258 | 0.003 |
| C6:0 | 0.435 | 0.515 | 12.164 | 0.002 | 0.168 | 0.685 |
| C8:0 | 0.681 | 0.416 | 8.671 | 0.006 | 8.236 | 0.008 |
| C10:0 | 2.149 | 0.154 | 12.024 | 0.002 | 1.923 | 0.176 |
| C11:0 | 18.286 | <0.001 | 1.707 | 0.202 | 0.209 | 0.651 |
| C12:0 | 0.280 | 0.601 | 0.817 | 0.374 | 0.125 | 0.727 |
| C13:0 | 13.289 | 0.001 | 1.315 | 0.261 | 1.320 | 0.260 |
| C14:0 | 4.801 | 0.037 | 14.838 | 0.001 | 0.000 | 0.983 |
| C15:0 | 0.256 | 0.617 | 8.786 | 0.006 | 1.305 | 0.263 |
| C16:0 | 0.035 | 0.852 | 8.531 | 0.007 | 0.018 | 0.895 |
| C17:0 | 0.127 | 0.724 | 25.479 | <0.001 | 3.958 | 0.056 |
| C18:0 | 0.323 | 0.574 | 6.622 | 0.016 | 0.996 | 0.327 |
| C20:0 | 2.289 | 0.142 | 3.036 | 0.092 | 2.017 | 0.167 |
| C21:0 | 4.913 | 0.035 | 3.092 | 0.090 | 5.446 | 0.027 |
| C22:0 | 0.360 | 0.554 | 1.634 | 0.212 | 7.776 | 0.009 |
| C23:0 | 0.481 | 0.494 | 6.260 | 0.018 | 18.787 | <0.001 |
| C24:0 | 0.641 | 0.430 | 49.405 | <0.001 | 14.633 | 0.001 |
| SFAs | 0.204 | 0.655 | 0.777 | 0.386 | 0.536 | 0.470 |
| MLCFAs | 0.043 | 0.838 | 2.742 | 0.109 | 12.881 | 0.001 |
