# Supplementary material for: rTMS ameliorates depressive‐like behaviors and regulates the gut microbiome and medium‐ and long‐chain fatty acids in mice exposed to chronic unpredictable mild stress
Source: CNS Neurosci Ther. 2023 Jun 2;29(11):3549–66. doi: 10.1111/cns.14287 (PMC10580350; doi:10.1111/cns.14287)
Supplement: Supplementary file 7 — Table S7 [file CNS-29-3549-s008.docx]

**Supplementary Table 7. Correlation between depressive-like behaviors and levels of MLCFAs in the hippocampus**

| Fatty acids | Distance in center (%) | | Sucrose preference rate (%) | | Immobility time (s) | |
| --- | --- | --- | --- | --- | --- | --- |
|  | r value | *P* value | r value | *P* value | r value | *P* value |
| C16:0 | -0.068 | 0.713 | -0.219 | 0.229 | 0.259 | 0.153 |
| C18:0 | 0.307 | 0.088 | 0.028 | 0.879 | -0.069 | 0.707 |
| C6:0 | 0.218 | 0.231 | -0.112 | 0.540 | -0.087 | 0.634 |
| C8:0 | 0.297 | 0.099 | 0.160 | 0.382 | -0.360 | 0.043 |
| C10:0 | 0.353 | 0.048 | 0.045 | 0.808 | -0.214 | 0.239 |
| C11:0 | 0.005 | 0.979 | -0.025 | 0.891 | 0.031 | 0.866 |
| C12:0 | 0.273 | 0.130 | -0.107 | 0.560 | -0.063 | 0.734 |
| C13:0 | -0.161 | 0.378 | -0.008 | 0.966 | 0.096 | 0.600 |
| C14:0 | 0.068 | 0.712 | -0.093 | 0.612 | -0.045 | 0.809 |
| C15:0 | -0.013 | 0.942 | 0.096 | 0.601 | -0.271 | 0.133 |
| C17:0 | 0.205 | 0.262 | 0.139 | 0.449 | -0.305 | 0.089 |
| C20:0 | -0.232 | 0.200 | -0.102 | 0.579 | 0.036 | 0.846 |
| C21:0 | -0.192 | 0.292 | -0.049 | 0.792 | 0.150 | 0.414 |
| C22:0 | -0.283 | 0.117 | 0.095 | 0.605 | 0.110 | 0.550 |
| C23:0 | -0.238 | 0.189 | -0.059 | 0.749 | 0.050 | 0.784 |
| C24:0 | -0.052 | 0.778 | 0.042 | 0.819 | -0.213 | 0.241 |
| SFAs | 0.123 | 0.502 | -0.185 | 0.310 | 0.195 | 0.284 |
| C24:1N9 | 0.089 | 0.627 | 0.270 | 0.135 | -0.128 | 0.484 |
| C18:1N9 | 0.472 | 0.006 | 0.284 | 0.115 | -0.303 | 0.092 |
| C14:1N5 | 0.019 | 0.916 | -0.122 | 0.505 | -0.239 | 0.188 |
| C15:1N5 | 0.078 | 0.672 | 0.183 | 0.316 | -0.005 | 0.978 |
| C16:1N7 | -0.002 | 0.990 | -0.260 | 0.151 | -0.142 | 0.437 |
| C17:1N7 | 0.133 | 0.470 | 0.143 | 0.435 | -0.167 | 0.362 |
| C18:1TN9 | 0.362 | 0.042 | 0.044 | 0.811 | -0.142 | 0.439 |
| C20:1N9 | -0.100 | 0.584 | 0.025 | 0.892 | 0.145 | 0.428 |
| C22:1N9 | 0.003 | 0.986 | 0.135 | 0.463 | -0.006 | 0.972 |
| MUFAs | 0.327 | 0.068 | 0.310 | 0.084 | -0.249 | 0.169 |
| C20:4N6 | 0.274 | 0.129 | 0.481 | 0.005 | -0.269 | 0.136 |
| C22:6N3 | 0.350 | 0.049 | 0.050 | 0.787 | -0.385 | 0.030 |
| C22:4N6 | -0.097 | 0.599 | 0.262 | 0.148 | -0.008 | 0.967 |
| C18:2N6 | 0.415 | 0.018 | 0.229 | 0.207 | -0.329 | 0.066 |
| C20:3N6 | 0.393 | 0.026 | 0.341 | 0.056 | -0.319 | 0.075 |
| C22:5N6 | 0.011 | 0.951 | 0.198 | 0.277 | -0.060 | 0.743 |
| C20:2N6 | 0.187 | 0.306 | 0.140 | 0.443 | -0.303 | 0.092 |
| C22:5N3 | 0.116 | 0.527 | 0.307 | 0.087 | -0.203 | 0.266 |
| C18:2TTN6 | 0.247 | 0.174 | -0.237 | 0.192 | -0.057 | 0.757 |
| C18:3N6 | 0.289 | 0.109 | 0.224 | 0.218 | -0.371 | 0.037 |
| C18:3N3 | 0.418 | 0.017 | 0.199 | 0.276 | -0.302 | 0.093 |
| C20:3N3 | 0.469 | 0.007 | 0.382 | 0.031 | -0.522 | 0.002 |
| C20:5N3 | 0.214 | 0.239 | 0.277 | 0.125 | -0.555 | 0.001 |
| C22:2N6 | 0.159 | 0.385 | 0.232 | 0.201 | -0.042 | 0.820 |
| PUFAs | 0.313 | 0.081 | 0.342 | 0.055 | -0.334 | 0.062 |
| Total MLCFAs | 0.367 | 0.039 | 0.305 | 0.089 | -0.264 | 0.145 |
