# Supplementary material for: rTMS ameliorates depressive‐like behaviors and regulates the gut microbiome and medium‐ and long‐chain fatty acids in mice exposed to chronic unpredictable mild stress
Source: CNS Neurosci Ther. 2023 Jun 2;29(11):3549–66. doi: 10.1111/cns.14287 (PMC10580350; doi:10.1111/cns.14287)
Supplement: Supplementary file 8 — Table S8 [file CNS-29-3549-s007.docx]

**Supplementary Table 8. Effect of CUMS and rTMS on the concentration of MLCFAs in the hippocampus**

| **Fatty acids** | **rTMS factor** | | **CUMS factor** | | **rTMS*CUMS** | |
| --- | --- | --- | --- | --- | --- | --- |
|  | F | *P* | F | *P* | F | *P* |
| C20:4N6 | 2.272 | 0.143 | 3.426 | 0.075 | 7.426 | 0.011 |
| C22:6N3 | 1.967 | 0.172 | 5.163 | 0.031 | 9.125 | 0.005 |
| C22:4N6 | 0.142 | 0.709 | 4.568 | 0.041 | 5.068 | 0.032 |
| C18:2N6 | 0.127 | 0.724 | 1.084 | 0.307 | 6.822 | 0.014 |
| C20:3N6 | 0.033 | 0.857 | 2.618 | 0.117 | 10.687 | 0.003 |
| C22:5N6 | 1.535 | 0.226 | 0.618 | 0.438 | 0.594 | 0.447 |
| C20:2N6 | 0.400 | 0.532 | 8.736 | 0.006 | 0.714 | 0.405 |
| C22:5N3 | 1.238 | 0.275 | 2.851 | 0.102 | 0.657 | 0.424 |
| C22:2N6 | 1.456 | 0.238 | 16.643 | <0.001 | 5.034 | 0.033 |
| C20:5N3 | 7.962 | 0.009 | 10.303 | 0.003 | 2.908 | 0.099 |
| C20:3N3 | 3.557 | 0.070 | 4.937 | 0.035 | 0.848 | 0.365 |
| C18:3N6 | 0.202 | 0.657 | 8.318 | 0.007 | 0.278 | 0.602 |
| C18:2TTN6 | 0.606 | 0.443 | 1.546 | 0.224 | 3.255 | 0.082 |
| C18:3N3 | 1.864 | 0.183 | 1.256 | 0.272 | 2.914 | 0.099 |
| PUFAs | 1.994 | 0.169 | 5.861 | 0.022 | 11.042 | 0.002 |
| C18:1N9 | 0.000 | 0.990 | 0.825 | 0.371 | 5.526 | 0.026 |
| C24:1N9 | 0.108 | 0.744 | 1.555 | 0.223 | 0.662 | 0.423 |
| C20:1N9 | 0.009 | 0.924 | 1.513 | 0.229 | 0.000 | 0.994 |
| C16:1N7 | 0.595 | 0.447 | 0.160 | 0.692 | 0.020 | 0.887 |
| C22:1N9 | 3.013 | 0.094 | 0.044 | 0.836 | 0.241 | 0.627 |
| C17:1N7 | 0.432 | 0.516 | 0.114 | 0.738 | 7.560 | 0.010 |
| C15:1N5 | 3.049 | 0.092 | 0.105 | 0.748 | 0.099 | 0.755 |
| C14:1N5 | 2.691 | 0.112 | 0.216 | 0.646 | 1.098 | 0.304 |
| C18:1TN9 | 0.056 | 0.815 | 2.488 | 0.126 | 1.393 | 0.248 |
| MUFAs | 0.065 | 0.800 | 0.162 | 0.690 | 3.111 | 0.089 |
| C6:0 | 2.886 | 0.100 | 5.609 | 0.025 | 0.452 | 0.507 |
| C8:0 | 0.004 | 0.951 | 4.023 | 0.055 | 0.511 | 0.481 |
| C10:0 | 0.704 | 0.409 | 0.929 | 0.343 | 5.698 | 0.024 |
| C11:0 | 0.622 | 0.437 | 0.359 | 0.554 | 2.609 | 0.117 |
| C12:0 | 4.684 | 0.039 | 2.050 | 0.163 | 0.000 | 0.993 |
| C13:0 | 1.426 | 0.242 | 1.690 | 0.204 | 1.281 | 0.267 |
| C14:0 | 0.104 | 0.749 | 0.500 | 0.486 | 0.046 | 0.831 |
| C15:0 | 0.605 | 0.443 | 2.655 | 0.114 | 5.522 | 0.026 |
| C16:0 | 0.697 | 0.411 | 2.723 | 0.110 | 0.219 | 0.643 |
| C17:0 | 0.530 | 0.473 | 9.775 | 0.004 | 2.132 | 0.155 |
| C18:0 | 2.181 | 0.151 | 3.401 | 0.076 | 0.758 | 0.391 |
| C20:0 | 0.179 | 0.675 | 0.534 | 0.471 | 0.927 | 0.344 |
| C21:0 | 1.866 | 0.183 | 4.397 | 0.045 | 0.163 | 0.690 |
| C22:0 | 0.021 | 0.885 | 2.918 | 0.099 | 0.023 | 0.880 |
| C23:0 | 0.288 | 0.596 | 0.286 | 0.597 | 0.154 | 0.698 |
| C24:0 | 0.595 | 0.447 | 1.466 | 0.236 | 0.727 | 0.401 |
| SFAs | 1.139 | 0.295 | 5.787 | 0.023 | 0.010 | 0.923 |
| MLCFAs | 0.092 | 0.765 | 0.617 | 0.439 | 2.916 | 0.099 |
