# Supplementary material for: rTMS ameliorates depressive‐like behaviors and regulates the gut microbiome and medium‐ and long‐chain fatty acids in mice exposed to chronic unpredictable mild stress
Source: CNS Neurosci Ther. 2023 Jun 2;29(11):3549–66. doi: 10.1111/cns.14287 (PMC10580350; doi:10.1111/cns.14287)
Supplement: Supplementary file 9 — Table S9 [file CNS-29-3549-s004.docx]

**Supplementary Table 9. Correlation between depressive-like behaviors and levels of MLCFAs in the prefrontal cortex**

| Fatty acids | Distance in center (%) | | Sucrose preference rate (%) | | Immobility time (s) | |
| --- | --- | --- | --- | --- | --- | --- |
|  | r value | *P* value | r value | *P* value | r value | *P* value |
| C16:0 | -0.036 | 0.843 | 0.012 | 0.950 | 0.315 | 0.079 |
| C18:0 | 0.225 | 0.215 | 0.085 | 0.645 | 0.326 | 0.069 |
| C17:0 | 0.363 | 0.041 | 0.299 | 0.097 | -0.049 | 0.789 |
| C20:0 | 0.027 | 0.884 | 0.277 | 0.124 | 0.043 | 0.816 |
| C14:0 | -0.131 | 0.475 | 0.133 | 0.467 | 0.118 | 0.522 |
| C22:0 | 0.165 | 0.368 | 0.209 | 0.250 | 0.099 | 0.588 |
| C24:0 | 0.038 | 0.837 | 0.185 | 0.312 | 0.079 | 0.667 |
| C15:0 | 0.328 | 0.066 | 0.406 | 0.021 | 0.119 | 0.516 |
| C23:0 | 0.015 | 0.936 | 0.186 | 0.309 | 0.307 | 0.087 |
| C21:0 | 0.082 | 0.657 | 0.233 | 0.200 | 0.315 | 0.079 |
| C8:0 | 0.142 | 0.439 | 0.451 | 0.010 | -0.048 | 0.796 |
| C12:0 | 0.044 | 0.811 | 0.169 | 0.354 | 0.151 | 0.410 |
| C10:0 | 0.036 | 0.845 | 0.104 | 0.571 | -0.086 | 0.639 |
| C6:0 | -0.183 | 0.316 | 0.133 | 0.466 | 0.072 | 0.695 |
| C13:0 | -0.163 | 0.374 | 0.216 | 0.235 | -0.025 | 0.892 |
| C11:0 | 0.027 | 0.884 | 0.354 | 0.047 | -0.074 | 0.687 |
| SFAs | 0.073 | 0.691 | 0.048 | 0.795 | 0.347 | 0.051 |
| C18:1N9 | 0.259 | 0.153 | 0.343 | 0.055 | 0.024 | 0.896 |
| C24:1N9 | 0.031 | 0.865 | 0.210 | 0.250 | 0.345 | 0.053 |
| C20:1N9 | -0.061 | 0.742 | 0.146 | 0.424 | 0.083 | 0.650 |
| C16:1N7 | 0.094 | 0.609 | 0.167 | 0.362 | -0.062 | 0.734 |
| C22:1N9 | -0.249 | 0.169 | 0.230 | 0.206 | 0.008 | 0.964 |
| C17:1N7 | 0.169 | 0.355 | 0.336 | 0.060 | -0.246 | 0.174 |
| C15:1N5 | -0.013 | 0.942 | 0.209 | 0.251 | 0.147 | 0.422 |
| C14:1N5 | -0.202 | 0.267 | 0.044 | 0.811 | 0.280 | 0.121 |
| C18:1TN9 | 0.029 | 0.875 | 0.031 | 0.865 | -0.072 | 0.695 |
| MUFAs | 0.159 | 0.383 | 0.335 | 0.061 | 0.244 | 0.178 |
| C20:4N6 | 0.240 | 0.185 | 0.306 | 0.089 | -0.207 | 0.256 |
| C22:6N3 | 0.178 | 0.330 | 0.309 | 0.085 | -0.333 | 0.062 |
| C22:4N6 | 0.107 | 0.561 | 0.277 | 0.125 | -0.202 | 0.267 |
| C18:2N6 | -0.034 | 0.855 | 0.410 | 0.020 | -0.126 | 0.491 |
| C20:3N6 | 0.158 | 0.387 | 0.427 | 0.015 | -0.350 | 0.049 |
| C22:5N6 | 0.015 | 0.933 | 0.355 | 0.046 | 0.176 | 0.335 |
| C20:2N6 | 0.109 | 0.553 | 0.417 | 0.017 | -0.129 | 0.481 |
| C22:5N3 | 0.034 | 0.852 | 0.400 | 0.023 | -0.129 | 0.482 |
| C22:2N6 | 0.303 | 0.092 | 0.145 | 0.429 | -0.301 | 0.094 |
| C20:5N3 | 0.213 | 0.242 | 0.279 | 0.123 | -0.403 | 0.022 |
| C20:3N3 | 0.192 | 0.292 | 0.240 | 0.186 | -0.212 | 0.243 |
| C18:3N6 | 0.067 | 0.716 | 0.325 | 0.069 | -0.002 | 0.989 |
| C18:2TTN6 | 0.175 | 0.337 | 0.324 | 0.071 | -0.005 | 0.980 |
| C18:3N3 | 0.402 | 0.023 | 0.279 | 0.122 | -0.106 | 0.562 |
| PUFAs | 0.215 | 0.237 | 0.368 | 0.038 | -0.267 | 0.140 |
| Total MLCFAs | 0.187 | 0.306 | 0.311 | 0.083 | 0.224 | 0.218 |
